# SEMA6C: a novel adhesion-independent FAK and YAP activator, required for cancer cell viability and growth

**DOI:** 10.1007/s00018-023-04756-1

**Published:** 2023-03-31

**Authors:** Damon Fard, Erika Testa, Valentina Panzeri, Sabrina Rizzolio, Giada Bianchetti, Virginia Napolitano, Silvia Masciarelli, Francesco Fazi, Giuseppe Maulucci, Bianca Maria Scicchitano, Claudio Sette, Maria Teresa Viscomi, Luca Tamagnone

**Affiliations:** 1 Department of Life Sciences and Public Health, Università Cattolica del Sacro Cuore, Rome, Italy; 2 Department of Neuroscience, Università Cattolica del Sacro Cuore, Rome, Italy; 3 Candiolo Cancer Institute, FPO-IRCCS, Candiolo, Turin, Italy; 4 Department of Anatomical, Histological, Forensic and Orthopaedic Sciences, Section of Histology and Medical Embryology, Sapienza University of Rome, Rome, Italy; 5 Fondazione Policlinico Gemelli-IRCCS, Rome, Italy

**Keywords:** Semaphorin, Signaling pathway, Kinase, FAK, YAP

## Abstract

Transmembrane semaphorins are signaling molecules, controlling axonal wiring and embryo development, which are increasingly implicated in human diseases. Semaphorin 6C (Sema6C) is a poorly understood family member and its functional role is still unclear. Upon targeting Sema6C expression in a range of cancer cells, we observed dramatic growth suppression, decreased ERK phosphorylation, upregulation of cell cycle inhibitor proteins p21, p27 and p53, and the onset of cell senescence, associated with activation of autophagy. These data are consistent with a fundamental requirement for Sema6C to support viability and growth in cancer cells. Mechanistically, we unveiled a novel signaling pathway elicited by Sema6C, and dependent on its intracellular domain, mediated by tyrosine kinases c-Abl and Focal Adhesion Kinase (FAK). Sema6C was found in complex with c-Abl, and induced its phosphorylation, which in turn led to FAK activation, independent of cell–matrix adhesion. Sema6C-induced FAK activity was furthermore responsible for increased nuclear localization of YAP transcriptional regulator. Moreover, Sema6C conferred YAP signaling-dependent long-term cancer cell survival upon nutrient deprivation. In conclusion, our findings demonstrate that Sema6C elicits a cancer promoting-signaling pathway sustaining cell viability and self-renewal, independent of growth factors and nutrients availability.

**Supplementary Information:**

The online version contains supplementary material available at 10.1007/s00018-023-04756-1.

## Introduction

Semaphorins are a large family of conserved extracellular signaling molecules, mediating cell–cell communication [1]. They were primarily described as axon guidance cues, but later implicated in the regulation of different biological processes, such as cardiovascular development, angiogenesis, bone homeostasis, and immune responses, as well as in their pathological counterparts [2]. In the tumor microenvironment, semaphorins modulate cancer cell growth, invasion and metastasis [3]; moreover, preclinical studies have highlighted certain family members as promising targets for cancer therapy [4].

Hitherto, more than 20 semaphorins have been described, comprising secreted, transmembrane, and cell surface-attached proteins, subdivided into eight classes based on structural features and sequence similarity. Transmembrane semaphorins can interact with surface molecules on neighboring cells (*in trans* signaling) or via shedding into the extracellular space; moreover, beyond this classical “forward” signaling, these semaphorins can use their cytoplasmic domain to deploy a so-called “reverse” signaling mode [5].

Semaphorin 6C (Sema6C) is a poorly studied family member, initially known as semaphorin Y [6]. It is a transmembrane molecule, considered a distal member in class-6, bearing a longer and partly divergent cytoplasmic domain. Actually, the signaling cascades elicited by this semaphorin, in either forward or reverse mode, are poorly known and await elucidation. Similar to other semaphorins, Sema6C was found to act as axon repelling and growth cone collapsing factor for DRG neurons. Intriguingly, Sema6C not only is expressed in neurons, but also in the embryonic targets: the myotome and dermatome; thus, it has been posited to regulate the navigation of extending axons, their halting in target areas, and synapse formation during development [6]. Interestingly, Sema6C expression in adult tissues is mostly localized to the brain and skeletal muscle [7], where it is suggested to play a role in neuromuscular junction stability [8]. Moreover, upon the re-activation of primordial ovarian follicles at puberty, the expression of Sema6C drops dramatically, suggesting its role in maintaining long-term cell survival in quiescence [9]. Intriguingly, Sema6C-binding antibodies have been recently shown to stimulate pancreatic carcinoma cell proliferation, possibly neutralizing a suppressor activity [10]; however the implicated mechanisms and relevance in cancer remain unclear. Moreover, high Sema6C levels strongly correlate instead with poor prognosis in multiple cancer types (e.g., colorectal, gastric, prostatic, endometrial, and ovarian carcinomas, based on public dataset analysis by Kmplot.com), indicating that the functional role and molecular signaling mechanisms of Sema6C in cancer cells need further investigation.

In the present work, we extensively analyzed Sema6C functional activity in diverse human cancer cells. Our data, based on gene knock-down, clearly indicate the requirement of Sema6C expression to sustain cancer cell proliferation, since Sema6C-depleted cells invariably underwent growth arrest, accompanied by autophagy induction and eventually cell senescence. In contrast, Sema6C overexpression elicited a novel signaling pathway sustaining long-term cancer cell viability upon growth factors- and nutrients-deprivation.

## Results

### Sema6C knockdown in cancer cells hampers proliferation, and elicits cellular senescence associated with increased autophagic flux

**Fig. 1:**
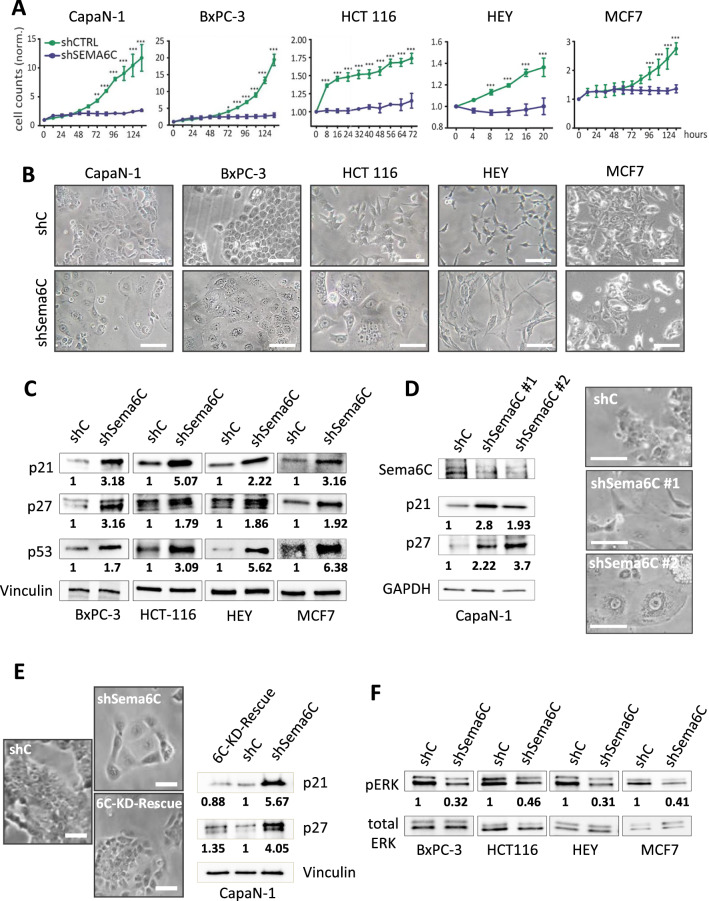
Sema6C knock-down leads to growth arrest and reduced ERK activity. **A** Representative time-course analyses of the growth in culture of the indicated cancer cells, representing diverse tumor types, either subjected to Sema6C silencing by targeted shRNA transfer (see Suppl. Fig. 1A) or transduced with control shRNAs (N = 3 replicates/group at each time point). Statistical significance was verified by two-way repeated measures ANOVA, with Bonferroni correction: *p < 0.05; **p < 0.01; ***p < 0.001. **B** Representative phase contrast micrographs of the cancer cell lines analyzed above. Scale bars: 50 μm. **C** Western blotting analysis and densitometric quantification of the expression of cyclin-dependent kinase inhibitors in the indicated control and Sema6C-silenced cancer cells; the images show representative results of consistent replicates. Vinculin levels provided a protein loading control, and were used in the normalization of band intensity analyses; the indicated values represent fold-changes in Sema6C-silenced cells vs. each respective control. **D** Western blotting analysis of Sema6C and cyclin-dependent kinase inhibitors in CapaN-1 cells subjected to Sema6C silencing by application of two independent targeted shRNAs (also see Suppl. Fig. 1B). GAPDH levels provided a protein loading control; band quantification values were calculated as described above. Similar analyses, performed in the other cancer cell models described above, are shown in Suppl. Fig. 1C, D. shRNA sequence #1 was commonly used in all other experiments in this study, where not specified. **E** CapaN-1 cells subjected to Sema6C knock-down (shSema6C) were further transferred with a cDNA construct forcing Sema6C re-expression (6C-KD-Rescue); 48 h later, representative phase contrast micrographs show reversion of the cellular phenotype in culture (comparable to that of control cells-shC, shown aside for reference). Scale bars: 50 μm. Western blotting analysis (on the right) shows reversion of p21/p27 upregulation upon Sema6C rescued expression; band quantification values were calculated as described for panel **C**. Similar analyses, performed in four other cancer cell models, are shown in Suppl. Fig. 2A–D. **F** Western blotting analysis of phosphorylated ERK levels in the indicated control and Sema6C-silenced cancer cells; the images show representative results of consistent replicates. Total ERK levels provided a protein loading control and were used in the normalization of band intensity analyses; the indicated values represent fold-changes in Sema6C-silenced cells vs. each respective control

In order to thoroughly elucidate the functional role and molecular signaling mechanisms of Sema6C in cancer cells, we assayed the impact of gene knock-down in different tumor cell types, such as pancreatic (CapaN-1, BxPC-3), colorectal (HCT116), ovarian (HEY), and breast (MCF7) human carcinoma cells. To this end, we applied Sema6C-targeted shRNA sequences, which consistently achieved up to 90% reduction of mRNA levels (Suppl. Fig. 1A). Interestingly, a few days after transferring shRNAs, we observed growth arrest and dramatic phenotypic changes in Sema6C-depleted cells (Fig. 1A, B), which acquired a flattened morphology compared to controls (transduced with an empty vector, shC). Cell cycle suppressor proteins p21, p27, and p53 were prominently upregulated in Sema6C-depleted cells, consistent with growth inhibition (Fig. 1C). The same effects were consistently induced by Sema6C targeting with two independent shRNA sequences (Fig. 1D and Suppl. Fig. 1B–D). Moreover, the specificity of gene silencing effects was confirmed by functional rescue experiments, by re-expressing Sema6C in cancer cells a few days after depletion of the endogenous protein (6C-KD-Rescue); this was sufficient to restore cancer cell growth and the parental phenotype, as well as to damp the upregulation of cell cycle inhibitors p21 and p27 (Fig. 1E, Suppl. Fig. 2A–D). Notably, one of the key drivers of cell cycle progression is ERK kinase, and its inactivation can cause cell cycle arrest and senescence by inducing p21 and p27 CDKIs [11]. We actually observed a significant decrease in (active) phospho-ERK levels upon Sema6C knock-down (Fig. 1F), indicating Sema6C-dependent regulation of ERK activity in cancer cells.

**Fig. 2:**
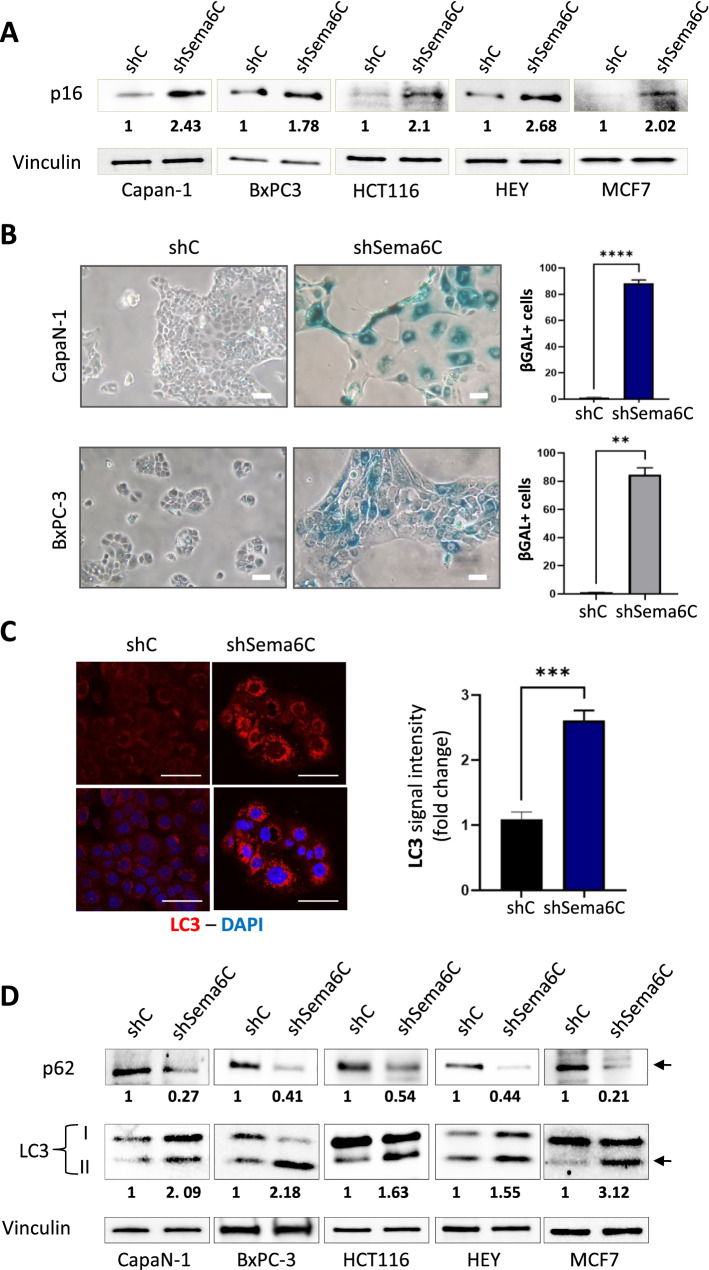
Sema6C knock-down is associated with increased autophagic flux and cancer cell senescence. **A** Western blotting analysis of the expression of senescent cell marker p16/INK4a in cancer cells representing different tumor types subjected to Sema6C silencing (shSema6C), or respective controls (shC); the images show representative results of consistent replicates. Vinculin levels provided a protein loading control, and were used in the normalization of band intensity analyses; the indicated values represent fold-changes in Sema6C-silenced cells vs. each respective control. **B** CapaN-1 and BxPC-3 cancer cells, either control or Sema6C-silenced, were incubated with the chromogenic substrate X-gal to reveal the activity of β-galactosidase, a typical marker of cellular senescence. Scale bars: 50 μm. The fraction of β-gal CapaN-1 and BxPC-3 positive cells is shown in the graphs on the right (N = 3 replicates/group). Unpaired t test with Welch’s correction: **p < 0.01; ****p < 0.0001. **C** Representative confocal images of the autophagic protein marker LC3 (red) in control or Sema6C-silenced BxPC-3 cells; nuclei were stained by DAPI. Scale bars: 50 μm. The bar graphs on the right shows the quantification of LC3 optical density fold-change upon Sema6C-depletion (average ± SD; N = 3 replicates/group). Unpaired t test with Welch’s correction: ***p < 0.001. Similar analyses, performed in four other cancer cell models, are shown in Suppl. Fig. 5A–D. **D** Western blotting analysis and densitometric values of p62 and LC3 autophagy markers in multiple cancer cells, either control or Sema6C-silenced; images show representative results of consistent replicate experiments. The degradation of p62 in association with prevalent increase of the lower LC3 band (LC3-II) is indicative of autophagy upregulation. Vinculin levels provided a protein loading control and were used in the normalization of band intensity analyses; the indicated values represent fold-changes of p62 and LC3-II levels in Sema6C-silenced cells vs. each respective control

Indeed, most cancer cells shortly died after the achievement of Sema6C silencing (Suppl. Fig. 3A), likely due to apoptosis, as indicated by PARP-1 protein cleavage (Suppl. Fig. 3B), and it was not possible to establish stable cell lines. However, some Sema6C-depleted pancreatic adenocarcinoma cells could be maintained in culture for a few weeks, and their phenotype was further analyzed. Notably, the latter appeared overtly larger in size, more circular and flattened on the plastic dish (Supp. Fig. 4A, B); moreover, cytofluorimetric analyses demonstrated that their actual volume was also significantly increased (Suppl. Fig. 4C). The phenotype consistently observed upon Sema6C knock-down was suggestive of a progressive establishment of cellular senescence [12–15]. This was confirmed by the upregulation of the senescence-associated protein p16 in cancer cells subjected to Sema6C silencing (Fig. 2A). Moreover, we observed a significant increase in the activity of the typical senescence-marker β-galactosidase [14] in Sema6C-depleted cells compared to controls (Fig. 2B). Notably, senescent cells maintain a metabolically active profile during growth arrest, which explains their progressive increase in size [16, 17].

Noteworthy, in senescent cells lysosomal function and numbers increase [18], which is associated with increased autophagic flux, possibly aimed at maintaining an active metabolic state. We thus assessed the autophagic flux in Sema6C-silenced cells using multiple approaches according to “Guidelines for detecting and monitoring autophagy” [19]. We tracked autophagosomes (APs) formation by LC3 immunostaining, and found that the latter increased in Sema6C-depleted cells compared to controls (Fig. 2C, Suppl. Fig. 5A–D). Moreover, we assessed by Western blotting the levels of active autophagosomal LC3-II isoform and of the autophagy substrate and cargo binding protein p62/SQSTM1, which gets degraded by the proteasome at the end of the autophagy cascade. Intriguingly, in Sema6C-depleted cells, the relative levels of LC3-II were higher than in control cells, while p62 was strikingly reduced (Fig. 2D). Thus, an adaptive upregulation of the autophagic flux correlates with growth inhibition and tumor cell senescence induced by Sema6C knock-down. Notably, based on transcriptomic analysis of human tumor samples, it emerges a significant correlation between Sema6C levels and autophagy-associated gene expression signatures (as defined in GSEA Molecular Signatures Database) (Suppl. Fig. 6).

### Sema6C overexpression sustains cancer cell viability upon nutrients and growth factors depletion, mediated by YAP activation

**Fig. 3:**
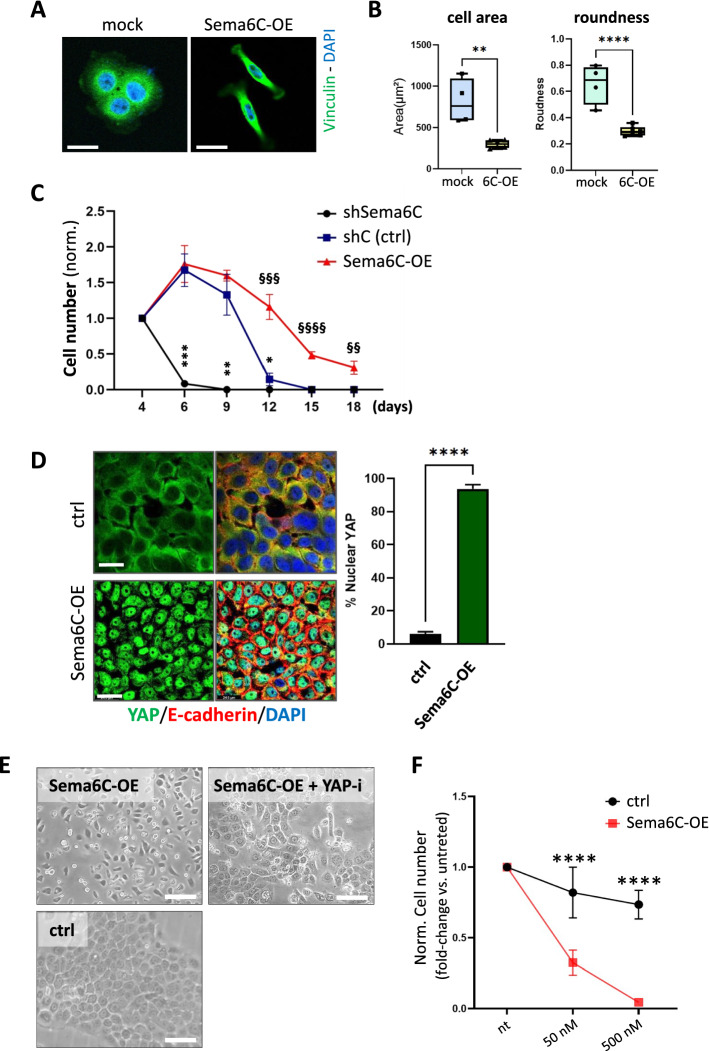
Sema6C overexpression in cancer cells elicited long-term survival and YAP activation. **A** Representative confocal images of BxPC-3 cells, either mock control or Sema6C-OE, immunostained with vinculin (green) and DAPI (blue). Scale bars: 20 μm. **B** Morphometric (Sholl) analysis of the cellular shape by Neurolucida software, applied to images of control or Sema6C-OE BxPC-3 cells (also see Suppl. Fig. 3B); representative plots of cell area and roundness quantification (N = 5 replicates for group; 50 cells/replicate). Unpaired t test with Welch’s correction: **p < 0.01; ****p < 0.0001. **C** BxPC-3 cells subjected to Sema6C knock-down, Sema6C-overexpression, or controls, were maintained in culture at confluence in serum-free conditions. The number of surviving cells in each condition was periodically scored and normalized to initial values (day 4); representative images for each time point are shown in Suppl. Fig. 10A (N = 3 replicates/group at each time point). By two-way repeated measures ANOVA (time × experimental condition), a significant effect of time (F(1.971, 11.83) = 129.5; p < 0.0001), of the experimental condition (F(2,6) = 350.5; p < 0.0001), and an interaction effect between time and experimental condition (F(10,30) = 39.0; p < 0.0001) were observed. Moreover, for a point-by point analysis comparing ShSema6C vs control or Sema6C-OE vs control, at the different time points, we performed multiple unpaired t-tests: ***p < 0.001; **p < 0.01 (shSema6C vs control); ^§§§§^p < 0.0001; ^§§§^p < 0.001; ^§§^p < 0.01 (OE-6C vs control). **D** Representative confocal images of mock-control and Sema6C-overexpressing BxPC-3 cells showing YAP (green) subcellular localization. Cell membranes were immunostained with E-cadherin (red) and nuclei were counterstained by DAPI. Scale bars = 50 μm. The graph bars on the right show the percentage of cells presenting nuclear YAP localization (N = 3 replicates/group at each time point). Unpaired t test with Welch’s correction: ****p < 0.0001. **E** Phase contrast micrographs of Sema6C-overexpressing BxPC-3 cells upon treatment with low concentration of the YAP pathway inhibitor Verteporfin (50 nM) or with DMSO vehicle only, for 24 h. Untreated mock-transduced control cells are shown below, for comparison. Scale bar = 50 µm. **F** Progressive viability loss of mock-transduced and Sema6C-overexpressing BxPC-3 cells cultured in serum-free medium (as in panel C) and treated with increasing verteporfin concentrations for 48 h (normalized to respective vehicle-only conditions) (N = 3 replicates/group at each time point). Two-way ANOVA: ****p < 0.0001

We stably enhanced Sema6C expression in cancer cells in order to elucidate the implicated signaling mechanisms. The cells immediately acquired a complementary phenotype to that observed upon gene silencing, looking smaller in size and characterized by loss of circularity and by the presence of polarized protruding processes (Fig. 3A, B, Suppl. Fig. 7A, B); moreover, they seemed to avoid cell–cell adhesion in culture. Intriguingly, we did not find any significant changes in the expression of epithelial-mesenchymal transition markers and transcriptional regulators upon Sema6C upregulation (Suppl. Fig. 7C), suggesting the involvement of distinctive molecular mechanisms to explain this phenotypic change. Cell motility assays actually revealed that Sema6C-overexpressing cells are more prone to spontaneous migration, in diverse experimental settings (Suppl. Fig. 8A–C). Notably, the expression of cell cycle inhibitors p21 and p27 was reduced in Sema6C overexpressing cells, complementary to what was observed upon gene silencing (Suppl. Fig. 9A), while pERK and phospho-mTOR levels were markedly induced by Sema6C (Suppl. Fig. 9A, B). The growth of Sema6C-overexpressing cells in highly permissive standard culture conditions showed a barely relevant increase compared to controls (Suppl. Fig. 9C). We then subjected BxPC3 cancer cells to nutrients- and growth factors-deprivation, by culturing in serum-free conditions for several days, without medium refreshing. Indeed, whereas in this setting control cells survived for up to 12 days, Sema6C-depleted cells revealed their frailty, undergoing cell death after 4 days (Fig. 3C, Suppl. Fig. 10A). On the other hand, Sema6C-overexpressing cells stood up to such metabolic stress up to 20 days. Notably, their survival was not associated with cellular senescence; in fact, after refreshing the culture with serum-completed medium on the last day, Sema6C-overexpressing cells resumed proliferation and attained confluency (Suppl. Fig. 10A). Similar results were observed by analyzing HCT-116 colon carcinoma cells (Suppl. Fig. 10B).

Intriguingly, cancer cell survival upon growth factor- and nutrient-deprivation has been associated with the activity of YAP transcriptional factor [20]. YAP/TAZ function is dependent on nuclear localization, which is induced by several pathways often related to mechanotransduction of extracellular signals. For instance, upon cell confluency and contact inhibition, YAP/TAZ nuclear localization is inhibited, and their accumulation in the cytosol leads to proteasome-mediated degradation [21]. Surprisingly, confocal analysis of Sema6C-overexpressing cells at confluency revealed YAP localization at nuclear level, whereas the same was expectedly mainly cytosolic in control cells (Fig. 3D). YAP nuclear localization was maintained by Sema6C expression also in serum-free conditions, indicating that this mechanism is independent from growth factors (Suppl. Fig. 11A). Moreover, western blotting analysis revealed Sema6C-dependent regulation of total YAP levels, commonly reliant on the proteasomal degradation of the cytosolic inactive fraction, in different cancer cell lines (Suppl. Fig. 11B). Altogether, these results suggested that Sema6C overexpression establishes constitutive YAP activation in cancer cells. In order to validate the functional role of YAP in Sema6C-induced cell phenotypes, we applied the commonly used YAP-inhibitor verteporfin. Notably, the treatment with very low concentrations of the inhibitor was sufficient to revert Sema6C-OE cell phenotype to that of controls (Fig. 3E). Moreover, Sema6C-overexpressing cells revealed their YAP-signaling dependence by quickly undergoing cell death upon verteporfin treatment in absence of serum, whereas control tumor cells showed significantly milder effects (Fig. 3F). Altogether, these data indicated that Sema6C elevated expression upholds YAP signaling in cancer cells, enabling YAP-dependent cell survival upon proliferative stress caused by growth factors- and nutrients-deprivation. Notably, higher Sema6C levels seemed to correlate with higher sensitivity to YAP inhibitor treatment in stringent conditions.

### Sema6C-induced regulation of cancer cells is dependent on Focal Adhesion Kinase

**Fig. 4:**
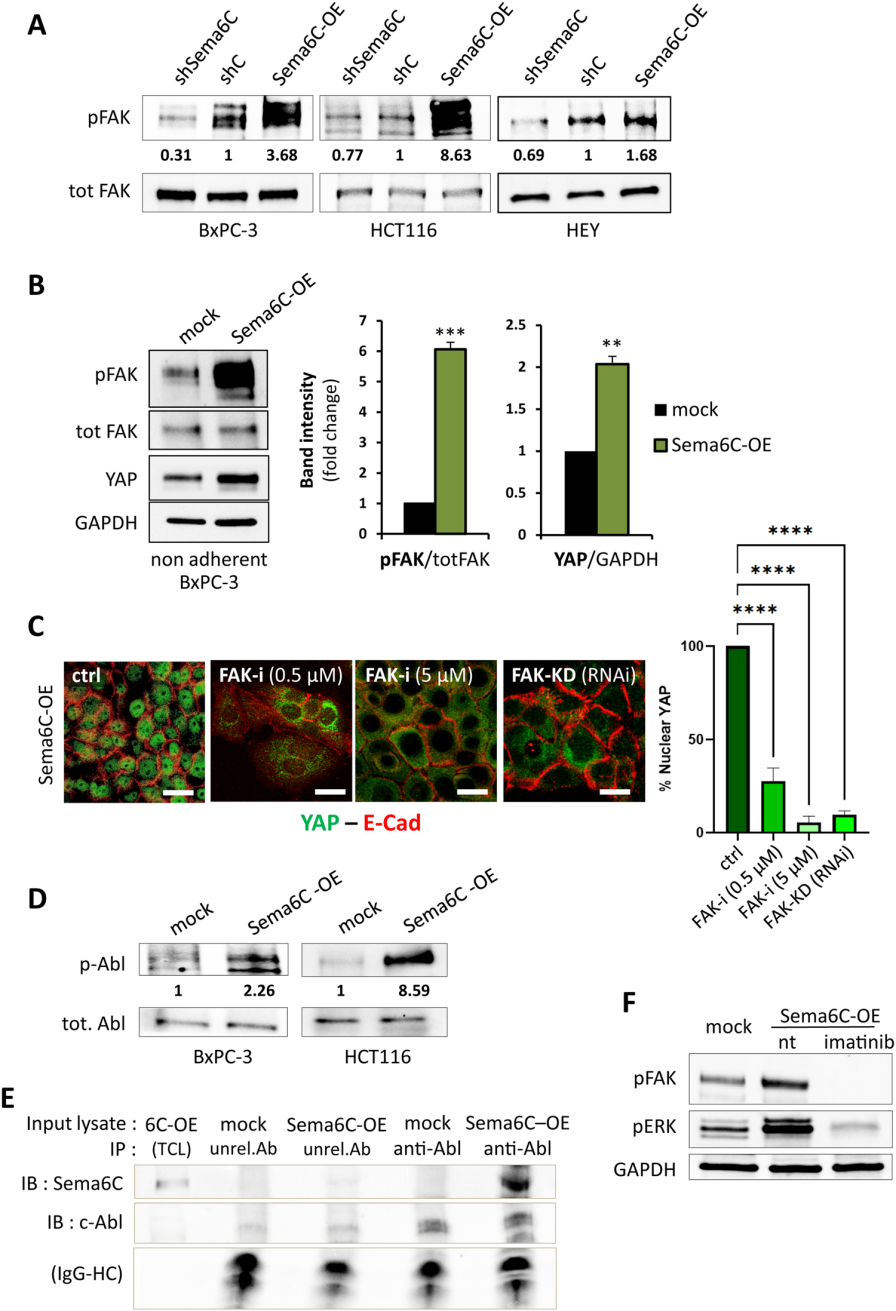
Sema6C-induced signaling cascade in cancer cells, mediated by c-Abl and FAK kinases. **A** Western blotting analysis and densitometric values of phosphorylated FAK protein in multiple cancer cells subjected to Sema6C knock-down, Sema6C-overexpression, or controls; images show representative results of consistent replicate experiments. Total FAK levels provided a protein loading control and were used in the normalization of band intensity analyses; the indicated values (N = 3 replicates/group) represent fold-changes in Sema6C-modulated cells vs. each respective control. **B** Western blotting analysis of phospho-FAK, total FAK, and YAP levels in detached Sema6C-overexpressing and control BxPC-3 cells in suspension; GAPDH provided a protein loading control. The image shows representative results consistently reproduced in two independent experiments, summarized in the graph on the right showing average values ± SD; statistical analysis was done by unpaired Student’s t test: **p < 0.01; ***p < 0.001. **C** Immunofluorescence confocal analysis of E-cadherin (red) and YAP subcellular localization (green) in Sema6C-overexpressing BxPC-3 cells treated with the FAK-inhibitor PF-573228 at concentrations 0.5 μM (for 16 h) or 5 μM (1 h), or subjected to FAK knock-down by shRNA transfection (see Suppl. Fig. 12C, D). Scale bars: 25 μm. The graph on the right indicates the cell fraction containing nuclear localized YAP (average values ± SD) (N = 3). Statistical analysis was done by two-way ANOVA: ****p < 0.0001. **D** Western blotting analysis and densitometric values of phosphorylated c-Abl protein in cancer cells bearing Sema6C-overexpression, or controls; images are representative of at least two independent replicas yielding consistent results. Total Abl levels provided a loading control and were used in the normalization of band intensity analyses; the indicated values (N = 3 replicates/group) represent fold-changes in Sema6C-OE cells vs. respective controls. **E** Co-immunoprecipitation experiments performed by incubating protein lysates of Sema6C-OE or mock control HCT116 cells with anti-Abl coated beads (or beads coated with a matched unrelated antibody, anti-ID3); the separated immunocomplexes were then probed to detect the presence of c-Abl kinase and any associated Sema6C protein. Detection of antibody heavy chains (IgG-HC) provided a control of immunocomplex loading. Data are representative of four independent experiments with consistent results. Similar results were obtained in BxPC3 cells, shown in Suppl. Fig. 15A. **F** Western blotting analysis of phosphorylated FAK and ERK proteins in Sema6C-OE BxPC-3 cells incubated (or not) with 10 μM imatinib for 2 h, and in mock cells for comparison. GAPDH levels provided a common loading control and were used in the normalization of band intensity analyses. Data shown are representative of two independent experiments with consistent results

We asked about signal transducers implicated in Sema6C-dependent activation of ERK, mTOR and YAP, accountable for the observed promotion of cancer cell growth and resistance to metabolic stress. Previous studies indicated Focal Adhesion Kinase (FAK) as an upstream activator of both mTOR and ERK pathways while its inhibition elicited cell cycle arrest [30–34] and cellular senescence [27, 28], as well as autophagy [29, 30]; moreover, YAP/TAZ activation was found to be induced downstream of FAK [31–34]. Actually, we found a striking and consistent correlation of FAK phosphorylation with Sema6C expression (Fig. 4A). Importantly, Sema6C-induced pFAK upregulation was independent of cell adhesion to the extracellular matrix, as assessed in detached cells in suspension (Fig. 4B), suggesting a novel mechanism of FAK regulation. Moreover, this correlated with cellular YAP protein accumulation, suggestive of its activation (Fig. 4B). Expectedly, the treatment of control cells with the selective FAK inhibitor PF-573228 resulted in a flattened and enlarged cell phenotype (Suppl. Fig. 12A), associated with p21 upregulation (Suppl. Fig. 12B), which was similar to that induced by Sema6C knock-down. On the other hand, the phenotype of Sema6C-overexpressing cells reverted to that of controls within one hour of treatment with PF-573228 (Suppl. Fig. 12C), and similar results were obtained upon FAK depletion by RNA interference (Suppl. Fig. 12D–E). Importantly, Sema6C-induced upregulation of YAP nuclear localization was also reversed upon FAK inhibition or knock-down (Fig. 4C), consistent with a Sema6C-FAK-YAP signaling cascade activated in these cells.

The relevance of FAK signaling and YAP activation downstream to Sema6C was further confirmed by transcriptomic analysis of human tumor samples, which revealed significant correlation of Sema6C levels with FAK- and YAP-regulated gene expression signatures (as defined in GSEA Molecular Signatures Database) (Suppl. Fig. 13). In sum, our data indicate a new signaling cascade driven by Sema6C, via FAK kinase, leading to pERK/mTOR and YAP activation in cancer cells.

### Sema6C-induced regulation of cancer cells depends on its intracellular portion and the associated c-Abl kinase

Transmembrane semaphorins like Sema6C are known to mediate both forward and reverse signaling, respectively dependent on the extracellular and intracellular portions of the molecule. In order to dissect this aspect, we initially tested the activity of a soluble recombinant Sema6C ectodomain in comparison to full-length Sema6C expressed in cancer cells (Suppl. Fig. 14A, B). The isolated ectodomain of Sema6C was totally ineffective in producing the phenotypic changes elicited by transmembrane Sema6C, implying that its reverse signaling is likely to be involved. Moreover, Sema6C ectodomain was unable to induce changes in pFAK and pERK levels (Suppl. Fig. 14C).

The intracellular portion of Sema6C contains motifs similar to those found in other class 6 semaphorins, and previously implicated in the interaction with non-receptor tyrosine kinases c-Src and c-Abl [35–38]; however, no intracellular interactors of Sema6C have been reported so far. Interestingly, we found that c-Abl phosphorylation was prominently increased in Sema6C overexpressing cells (Fig. 4D), suggesting a regulatory mechanism. Moreover, by immunoprecipitation experiments in Sema6C-OE cells, we observed a clear and specific interaction between Sema6C and endogenous c-Abl tyrosine kinase (Fig. 4E, Suppl. Fig. 15A). Of interest, lines of evidence have indicated that c-Abl can regulate the activity of FAK kinase [39]. This signaling cascade impacts on cytoskeletal dynamics, cell shape, cell–cell and cell–matrix connections, but could furthermore regulate cell proliferation and survival through mechanotransduction pathways like Hippo-YAP/TAZ. Thus, we wondered about the role of c-Abl upstream the signaling cascade elicited by Sema6C. Indeed, upon inhibition of Abl tyrosine kinase with imatinib, the phenotype of BxPC3 Sema6C-OE cells reverted to control in 2 h (Suppl. Fig. 15B). Moreover, Abl inhibition prevented the activation of newly described Sema6C effectors FAK, ERK and YAP (Fig. 4F, Suppl. Fig. 15C). These data support the identification of a novel Sema6C-induced signaling pathway accountable for phenotypic and functional changes in cancer cells.

## Discussion

Beyond their role in embryo development, semaphorins are emerging as pivotal regulators of tumor progression [40]. Interestingly, transmembrane semaphorins are bidirectional signals, which mediate both conventional “forward” and peculiar “reverse” cell–cell communication [5]. Yet, the intracellular domain of these semaphorins is poorly conserved, and the implicated molecular mechanisms are mostly unknown. In this work, we focused on Sema6C, a poorly studied transmembrane semaphorin that was first discovered as an axonal chemorepellent [6], enriched in skeletal muscles and neuromuscular junctions [8], and implicated in ovarian follicles maturation [41], while its role in cancer context remains unclear. Our data, validated with multiple approaches, in diverse tumor cell types, are consistent with a growth-promoting activity of Sema6C, mediated by a novel signaling cascade in cancer cells, culminating in the functional activation of ERK and YAP effector proteins. In fact, while Sema6C expression knock-down consistently caused growth arrest and cancer cell senescence, its overexpression conferred independence from serum-borne growth factors and nutrients.

Sema6C, like other semaphorins, has been found to inhibit axonal extension; however, we have not observed inhibitory effects mediated by Sema6C in cancer cells. This is not surprising, since divergent semaphorin functions in neurons and in cancer cells are commonly seen (e.g. for Sema3A, Sema3E, Sema4D, etc.), likely reflecting the involvement of different receptor complexes and signaling pathways [1, 42, 43]. Noteworthy, during the preparation of this manuscript, Hung and colleagues reported that a Sema6C-targeted antibody promoted the proliferation of pancreatic carcinoma cells [10]; such findings led the authors to conclude on a tumor-suppressor activity of this semaphorin, seemingly in conflict with multiple lines of evidence presented here. Actually, in our study we have widely analyzed the functional and signaling impact of Sema6C overexpression in diverse cancer cells, never detecting signs of growth inhibition, and instead finding evidence of enhanced cell viability vs. controls, upon growth factor- and nutrient-deprivation, not to mention the activation of YAP signaling. Moreover, Sema6C knock-down in cancer cells of diverse origin invariably led to cell cycle arrest and cellular senescence, which is rather consistent with a role of this semaphorin in sustaining the cell cycle. One possible explanation for this inconsistency of our data with those reported by Hung et al. is a distinctive function of Sema6C in a subset of pancreatic cancer cells. Future work will elucidate whether Sema6C-targeted antibodies may actually trigger, rather than blocking, the reverse signaling cascade illustrated in our study.

Intriguingly, cell motility assays revealed that Sema6C-overexpressing cells are more prone to spontaneous migration, in diverse experimental settings. We did not find any significant changes in the expression of epithelial–mesenchymal transition markers or transcriptional regulators upon Sema6C upregulation, suggesting the involvement of distinctive molecular mechanisms to explain this phenotypic change. It is tempting to speculate, based on previous literature, that Sema6C-dependent FAK kinase activation may be involved in increased cancer cell motility. Although these findings may not be strictly related to the regulation of cell viability and proliferation by Sema6C, described in this paper, we believe they are particularly relevant and deserve further investigation in future studies.

While Sema6C signaling mechanisms are poorly understood, here we show for the first time that this semaphorin controls the phosphorylation and functional activation of major intracellular signal transducers in cancer cells: FAK, ERK and mTOR kinases. FAK protein is often overexpressed in advanced human malignancies, and it is considered a signaling hub in cancer cells, controlling a spectrum of biological functions, from cell shape remodeling, to cell migration and metabolism [44]. Conversely, FAK inhibition has been associated with reduced cancer cell proliferation and the induction of autophagy and senescence [28, 30, 49], with the upregulation of p21, p27, and p53 cell cycle inhibitor proteins [28, 49], and with the loss of YAP nuclear localization [33]. Here we show that Sema6C expression is strikingly correlated with the level of phosphorylated active FAK in cancer cells. Notably, FAK is known to become activated in integrin-based focal adhesions, dependent on cell–matrix interaction and cytoskeletal regulation [34]. However, interestingly we found that Sema6C-induced FAK phosphorylation is independent of cell–matrix adhesion, as indicated by the analysis of detached cancer cells in suspension; which implicates a novel mechanism of FAK regulation governed by Sema6C reverse signaling.

FAK activity was reported previously to promote both mTOR and ERK signaling [30, 45–47]; moreover, accumulating evidence implicates FAK as a key upstream activator of YAP/TAZ signaling in cancer cells [44, 48]*.* For instance, a FAK-YAP-mTOR signaling cascade controls stem cell-based tissue regeneration in mice [48]. Our experimental evidence indicated that Sema6C promotes YAP protein increase and nuclear localization, independent of cell–cell contact regulation and cell–matrix adhesion, while YAP signaling blockade suppressed Sema6C-induced phenotype in cancer cells. Notably, by the treatment with a selective inhibitor (or achieving gene knock-down), we could place FAK activity upstream of Sema6C-induced phenotypic changes and YAP nuclear localization in cancer cells, potentially reflecting a novel mechanism in control of YAP activity. The general relevance of our experimental data is supported by the observation that Sema6C expression is highly significantly correlated with FAK signaling and YAP-induced transcriptional signatures in human tumor samples.

**Fig. 5:**
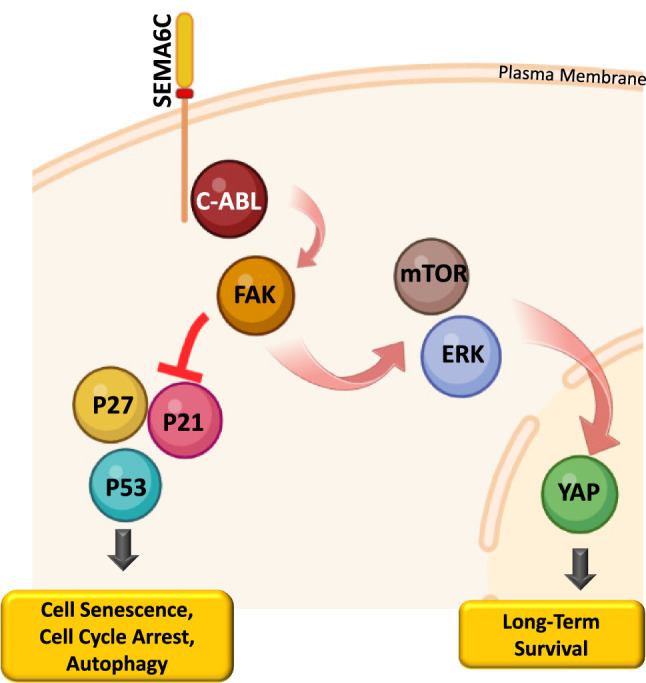
Schematic representation of Sema6C signaling cascade

Interestingly, the phenotype produced by Sema6C silencing was not rescued by the treatment with a soluble extracellular domain of the semaphorin (data not shown). Moreover, the overexpression of Sema6C ectodomain in cancer cells did not replicate the phenotypic changes elicited by transmembrane Sema6C, likely implicating reverse signaling mechanisms triggered by the intracellular tail, that were never investigated previously for this semaphorin. Experiments assaying the physical association between FAK and Sema6C were inconclusive (not shown). However, other semaphorin members of class 6, such as Sema6A and Sema6D, have been shown to recruit non-receptor tyrosine kinases to their intracellular domains [38, 50]. In this study, we unveiled that Sema6C recruits c-Abl in a complex, which upregulates its kinase activity. Hence, Sema6C-induced activation of c-Abl drives FAK phosphorylation, which is consistent with previous evidence linking the two intracellular kinases [51], and could potentially represent the initial step of a novel Sema6C signaling cascade in cancer cells (summarized in Fig. 5).

Since multiple tumor cells proved to depend on Sema6C signaling for proliferation, and its high expression promoted an aggressive phenotype in culture, our data support the idea that Sema6C-targeting may have beneficial effects for cancer therapy. Moreover, cancer cells overexpressing Sema6C upheld constitutive YAP activation and signaling dependence, a potential liability of interest for therapy. Other aspects need to be investigated further. For instance, it is unknown the potential impact of Sema6C-inhibitors in normal adult tissues. One intriguing element in this respect is that Sema6C knock-out mice are reported to be viable and fertile (MGI:3604574). It may be therefore hypothesized that, different from what we observed in cancer cells, Sema6C expression is not essential during development, being compensated by redundant functions of other family members, which may support future experiments aimed at systemic Sema6C-targeting in mouse cancer models.

In conclusion, our data indicate that human tumor cells are dependent on Sema6C expression for viability and growth, identifying a novel potential target for cancer therapies. Moreover, we unveiled a previously unknown Abl kinase-dependent retrograde signaling pathway mediated by transmembrane Sema6C, upregulating FAK, ERK and YAP activity in cancer cells, and conferring refractoriness to cell cycle inhibitory mechanisms.

## Materials and methods

### Cell culture and reagents

Cell lines (BxPC-3 and CapaN-1 pancreatic adenocarcinoma, HCT-116 colorectal adenocarcinoma, HEY ovarian carcinoma, MCF7 breast luminal carcinoma) were grown in a monolayer at 37° C in 5% CO2 atmosphere. BxPC-3 and HEY were cultured in RPMI-1640 medium supplemented with 10% FBS; HCT-116 and MCF-7 in DMEM medium with 10% FBS; and Capan-1 in Iscove medium with 20% FBS. Culture media were supplemented with 2 mM glutamine, 100U/mL penicillin, and 100 μg/mL streptomycin.

### Antibodies and other reagents

Anti-Sema6C antibodies were from R&D Company (Cat. #AF2108). The following antibodies were from Cell Signaling Technology: anti FAK (Cat. #3285), anti pFAK (Y925) (Cat. #3284), anti-LC3A/B (Cat. #4108), anti-phospho p44/42 MAPK (Erk1/2, 137F5) (Cat. #4695), anti-P44/42 MAPK (Erk1/2, 137F5), anti-p21 Waf1/Cip1 (Cat. #2947), anti-p27 Kip1 (#2552), anti-PARP1 (#9542S), anti-p16/INK4a P16 (Cat. #80772), anti-SQSTM1/p62 (Cat. #5114), anti-phospho-c-Abl (Tyr245) (Cat. #2861). Santa Cruz Biotechnology provided: anti c-Abl (8E9) (Cat. #sc-56887), anti p53 (DO-1) (Cat. #sc-126), anti-YAP (63.7) (Cat. #sc-101199), anti-GAPDH (6C5) (Cat. #sc-32233). Anti-vinculin was from Sigma-Aldrich Company (Cat. #V9131), anti-E-cadherin from Proteintech (cat. #20874-1-AP). Moreover, PF-573228 (Tocris Bioscience Cat. #3239), Verteporfin (Sigma-Aldrich; Cat. #SML0534), Imatinib (Cell signaling, Cat. #9084).

### Gene knock-down and overexpression

For SEMA6C knock-down, we applied the following shRNA-expressing constructs provided by Sigma-Aldrich: TRCN0000005666 (indicated as shSema6C#1, applied in most experiments) and TRCN0000005664 (shSema6C#2). To achieve FAK/PTK2 knock-down, we applied the following shRNA-expressing constructs provided by Sigma-Aldrich: TRCN0000121207 (indicated as #1, and commonly used in shown experiments); TRCN0000196310 (#2); TRCN0000121318 (#3); TRCN0000194984 (#4). To achieve SEMA6C overexpression, the respective human cDNA was produced by gene synthesis by BioCat GmbH (Heidelberg, Germany), and subcloned in pLVX-puro lentiviral transfer plasmid (carrying puromycin resistance). Sema6C-Fc construct (Plasmid: #72167 from Addgene repository) was subcloned into the same lentiviral transfer plasmid.

### Gene transfer with lentiviral vectors

HEK-293T packaging cells were used to produce non-replicating viral particles with shRNAs or cDNA expressing constructs. As previously described, cell supernatants containing lentiviral particles were incubated with cultured cells in the presence of polybrene 8 μg/ml. [52]. Cells transduced with vectors containing puromycin resistance were consequently selected in the presence of 1 μg/ml puromycin in the medium.

### mRNA expression analysis

The RNeasy Mini kit (Qiagen) was used to get total mRNA extracts. In a final volume of 20 μL, 1 μg of RNA was retrotranscribed using the Improm-II Reverse Transcription Set (Promega), according to the manufacturer’s protocol. Real-time PCR was used to evaluate gene expression using specific SYBR Green primers provided by Sigma-Aldrich Company (see table below). According to the formula below, the fold change was calculated: Fold increase = 2^-(CT of target gene − CT of the house-keeping gene). Primers applied were as follows:

**Table:** 

h-Sema6C-S	5′-CTTCGGCTCAACTGCTCTGT
h-Sema6C-AS	5′-AACCCACGCTCAATCTCATC
h-GAPDH-S	5′-TTGTTGCCATCAATGACCC
h-GAPDH-AS	5′-CCTCCCGTTCTCAGCCTTG

### Protein immunoprecipitation and Western blotting analysis

Cells were lysed in TritonX100-containig LB buffer, in presence of PMSF (phenylmethylsulfonyl fluoride), and Protease Inhibitor Cocktail (all provided by Cell Signaling Technology). Bradford assays were used to determine protein concentration in cell lysates. For the analysis of total cell lysates, equal amounts of total proteins were denatured by boiling in Laemmli buffer (2% SDS, 50 mM Tris–HCl, pH 7.4, 20% mercaptoethanol, and 20% glycerol) and analyzed by SDS-PAGE and Western blotting, according to standard methods and manufacturers’ instructions. For protein immunoprecipitation, equal amounts of cell lysates were incubated with Protein G-Sepharose beads (Abcam company #ab193259) coated with specific antibodies for 2 h at 4 °C with. After rinsing the beads, the immunoprecipitated proteins were denatured by boiling in Laemmli buffer and subjected to SDS-PAGE and Western blotting analysis.

### Cell senescence assay

Cells were seeded in 6-well plates and upon reaching 50–60% confluency, the medium was removed. The cells were washed in PBS before being fixed with the 1 × fixative solution provided with the senescence-galactosidase staining kit (Cell Signaling Technology; #9860). Following the manufacturer’s instructions, a fresh beta-galactosidase staining solution was prepared. After washing with PBS twice, cells in each well were stained with a 1 mL staining solution. After 16-h incubation at 37 °C in a dry incubator, senescent cells were identified for a positive beta-galactosidase-dependent dye conversion.

### Crystal violet staining

Cells growing in 6-well culture dishes were rinsed with PBS and fixed in 4% PAF for 15 min; after further washes with PBS, the cells were incubated with crystal violet staining solution (Sigma, cat. V5265) for 10–15 min, and finally rinsed 3 times with PBS/water before light microscopy analysis.

### Immunofluorescence and confocal analysis

Cells were seeded on glass coverslips, then fixed for 15 min in 4% paraformaldehyde, permeabilized with (0.1% or 0.3%, as indicated in specific experiments) Triton/phosphate-buffered saline for 10 min at room temperature, and blocked by incubation with 5% normal donkey serum for 30 min or 10% BSA for 1 h. After incubation with primary antibodies overnight and subsequent rinses, fluorochrome-conjugated secondary anti-mouse or anti-rabbit antibodies were added for 1 h at room temperature. 4,6-diamidino-2-phenylindole (DAPI) was used to reveal cell nuclei. The coverslips were finally washed and mounted on slides. Images were acquired at room temperature with a confocal laser scanning microscope (CLSM SP5 Leica) equipped with a 63 × /1.30 HCX Plan-Apochromat oil immersion objective lens (ACS APO 63 × /1.30 oil CS 0.17/E, 0.16). Confocal settings were maintained constant throughout the acquisition of slides from the different experimental groups. Images were analyzed off-line with LAS AF Lite software. Morphological analysis was performed by using a fluorescence microscope (Zeiss) equipped with a motorized stage and a camera connected to software (Neurolucida 7.5, MicroBright-Field, Germany) that allows a 3D quantitative analysis of the cells.

### Scholl morphometric analysis of cellular phenotype

Cells were imaged using a fluorescence microscope (Zeiss) equipped with a motorized stage and a camera connected to software (Neurolucida 7.5, MicroBright-Field) that allowed a quantitative 3D analysis of the entire compartment. Only cells that displayed a clear and intact nucleus were included in reconstructions. Fifteen cells per group were randomly selected and included for analysis. Sholl analysis of the cell soma was performed for each cell.

### Cell growth analysis and cytotoxicity assay

Cells were seeded in a 96-well plate (2000 cells/well), cultured into IncuCyte SX5 Live-content imaging system (Essen Bioscience) at 37 °C with 5% CO_2_ atmosphere, and imaged at 10 × magnification. Three wells (four fields/well) for each condition were analysed using IncuCyte software, counting adherent cells labeled with Nuclight Rapid NIR. Cell numbers at each time point were normalized to those seeded at time *T*_0_. For cytotoxicity analysis, the cells (5000/well) were labeled with the Cytotox Green Dye (Sartorius) to detect dying cells, and with Nuclight Rapid NIR (Sartorius) to detect the nuclei of all adherent cells. The images (4 images/well) were analysed using IncuCyte software to detect dying cells labelled with Cytotox Green Dye. The number of dying cells was represented as ratio of Cytotox Green Dye labelled cells to Nuclight Rapid NIR labelled cells.

### Cellular survival assays

Cell survival upon serum-starvation was similarly analyzed as follows. A fixed number of cells was seeded in six-well dishes; at the specific time points, the cells were fixed in 4% PAF and counted by taking images in five non-overlapping random-selected microscopy fields. A minimum of three replicates for experiment were performed. Cell counts in replicas of each experimental condition were averaged, calculating fold changes over cell numbers at the initial time point.

### Cell size analysis by cytofluorimetry

Cells harvested by trypsinization cells (2 × 10^5^ per condition) were washed once in PBS to remove the culture medium, and then resuspended in 300 μl PBS. Cell dimension was analyzed right afterward by flow cytometry, excluding from the analysis the dead cells, on the basis of the forward/side scatter flow cytometer parameters.

### Cell migration assays and time lapse analysis of cellular motility

For time-lapse imaging of spontaneous motility, cells seeded on a µ-Slide 8 well (ibidi GmbH, Grafelfing, DE), at a concentration of 5000 cells/well, were first fluorescently labeled by incubation with CellTracker™ Green CMFDA Dye (Molecular Probes, Inc, Eugene, OR, USA) for 1 h at 37 °C-5% CO_2_. The cells in culture were then imaged by a Nikon A1-MP confocal microscope (Nikon Instruments, Inc., Melville, NY), equipped with an on-stage incubator (OKOLAB). Intensity images were collected with a 20 × objective (1.4 NA) and a large image composed of 1946 × 1946 pixels (604.7 × 604.7 µm) was obtained for each sample. Start positions of individual samples were registered as an array of *XY* coordinates of the stage position using the NIS-Elements software (Nikon Instruments, Inc.). Images were taken from each *XY* position of the array every 3 min and 9 s for a total time-lapse acquisition of 4 h. Cells movement were analysed using the Cell Motility plugin of NIS-Elements software. The program recognizes cell centroids automatically and continually records their motions, producing quantitative statistics such as cell *XY* coordinates, velocities, and trajectory lengths. The average speed and the average path length of 50 randomly selected cells from each sample were evaluated and compared to assess differences between groups. Statistical tests for sets of biological/biophysical data were performed by R Studio (https://www.rstudio.com/); baseline characteristics among groups were compared with Student’s t-test.

For Boyden chamber-like migration assays, we used Transwell inserts with 8 μM pore size (Corning Costar Incorporated, NY, USA). In brief, the lower side of the semipermeable member was precoated with 10 µg/ml fibronectin and blocked with 1% BSA; then 5 × 10^4^ cells resuspended in serum-free medium were included in the upper chamber and allowed to migrate through the filter separating the bottom chamber, which contained 1% FBS-containing medium. After 16 h of incubation, non-migrated cells on the upper side of the filter were removed with a cotton swab. The insert was then fixed with 4% paraformaldehyde and stained with crystal violet. The integrated pixel values of light microscopy images of the inserts were then measured using ImageJ (NIH). Experiments were replicated at least three times in order to ensure results consistency.

For monolayer wound healing assays, the cells were grown in a six-well cluster (Corning Costar Incorporated, NY, USA) until forming a confluent monolayer. After 24 h, the cellular monolayer was scratched by a 1000 µl pipette tip, washed with PBS, and overlaid with fresh medium, taking a first microscopy image at *T*_0_. The dish was then placed in a cell culture incubator at 37 °C, allowing cell migration to fill the gap. After 24 h, the cell monolayer was washed with PBS, fixed with 4% paraformaldehyde, and finally stained with 1% crystal violet. Images were acquired by phase-contrast microscopy and analyzed quantitatively by ImageJ software. The percentage of wound closure at the end of the experiment was assessed by overlaying the scratched area in *T*_0_ images, for each condition.

### Statistical analysis

All values were expressed as mean ± SD. Differences between means were analyzed by using *t* test or One-way ANOVA, Two-way (multiple groups) or repeated-measure analysis of variance (ANOVA) followed, in cases of significance, by a Bonferroni post hoc test was applied. See figure legends for more details. Values of p ≤ 0.05 were considered to be statistically significant. Statistical analyses were carried out by GraphPad Prism 6 (GraphPad software for Science, San Diego, CA).

## Supplementary Information

Below is the link to the electronic supplementary material.

Supplementary file1 (MP4 997 KB)

Supplementary file2 (MP4 1012 KB)

Supplementary file3 (PDF 7247 KB)

## Data Availability

Data generated or analysed during this study are mostly included in this published article and its supplementary information files (additional raw data analysed in the current study are available from the corresponding author on reasonable request).
