# Supplementary material for: SEMA6C: a novel adhesion-independent FAK and YAP activator, required for cancer cell viability and growth
Source: Cell Mol Life Sci. 2023 Mar 31;80(4):111. doi: 10.1007/s00018-023-04756-1 (PMC10066115; doi:10.1007/s00018-023-04756-1)
Supplement: Supplementary file 3 — Supplementary file3 (PDF 7247 KB) [file 18_2023_4756_MOESM3_ESM.pdf]

Suppl. Fig. 1

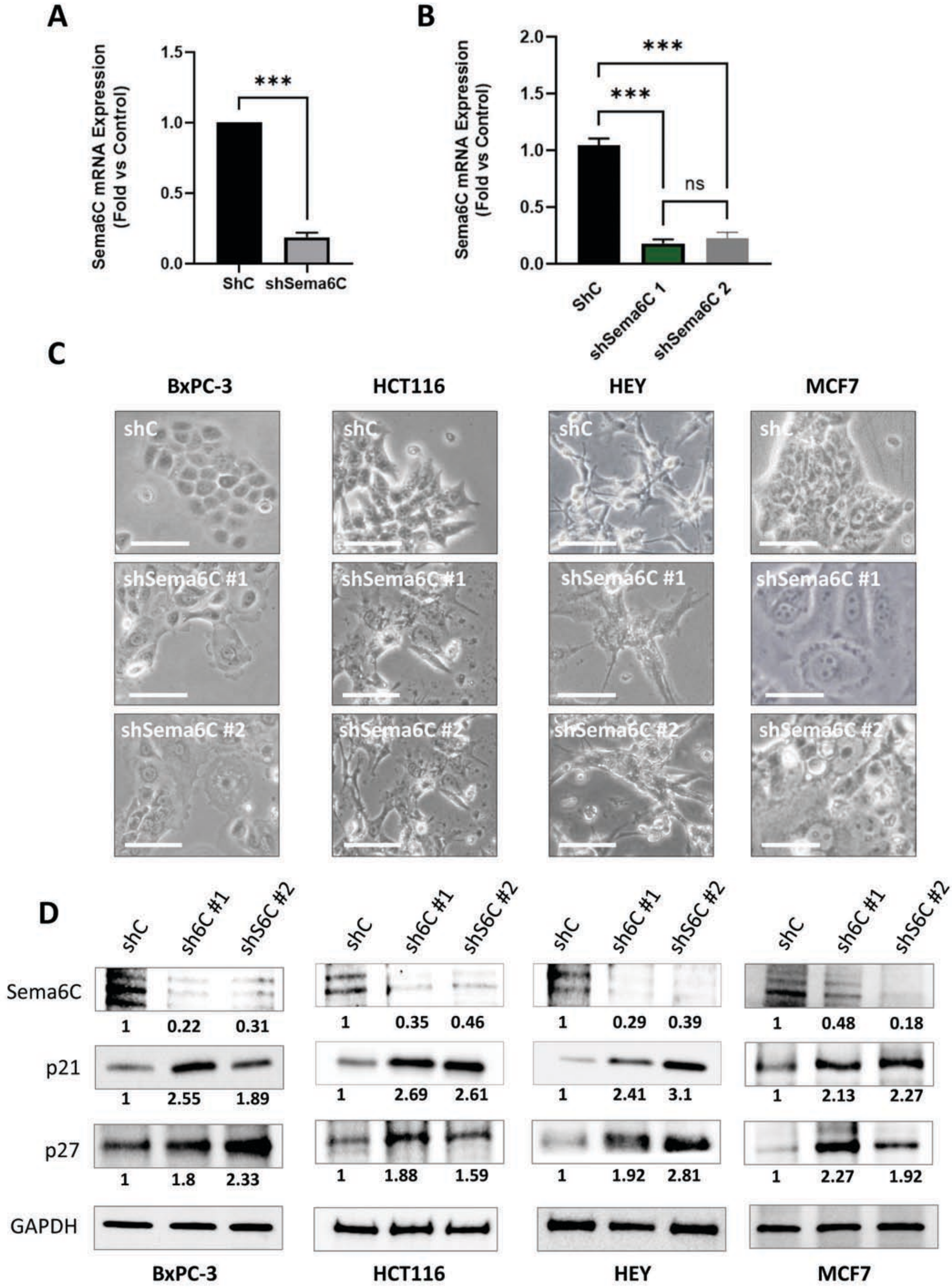
A
Sema6C mRNA Expression (Fold vs Control)
1.5
1.0
0.5
0.0
***
ShC
shSema6C
B
Sema6C mRNA Expression (Fold vs Control)
2.0
1.5
1.0
0.5
0.0
***
***
ns
ShC
shSema6C 1
shSema6C 2
C
BxPC-3
HCT116
HEY
MCF7
shC
shSema6C #1
shSema6C #2
D
shC
sh6C #1
shS6C #2
Sema6C
1 0.22 0.31
1 0.35 0.46
1 0.29 0.39
1 0.48 0.18
p21
1 2.55 1.89
1 2.69 2.61
1 2.41 3.1
1 2.13 2.27
p27
1 1.8 2.33
1 1.88 1.59
1 1.92 2.81
1 2.27 1.92
GAPDH
BxPC-3
HCT116
HEY
MCF7

Suppl. Fig. 2

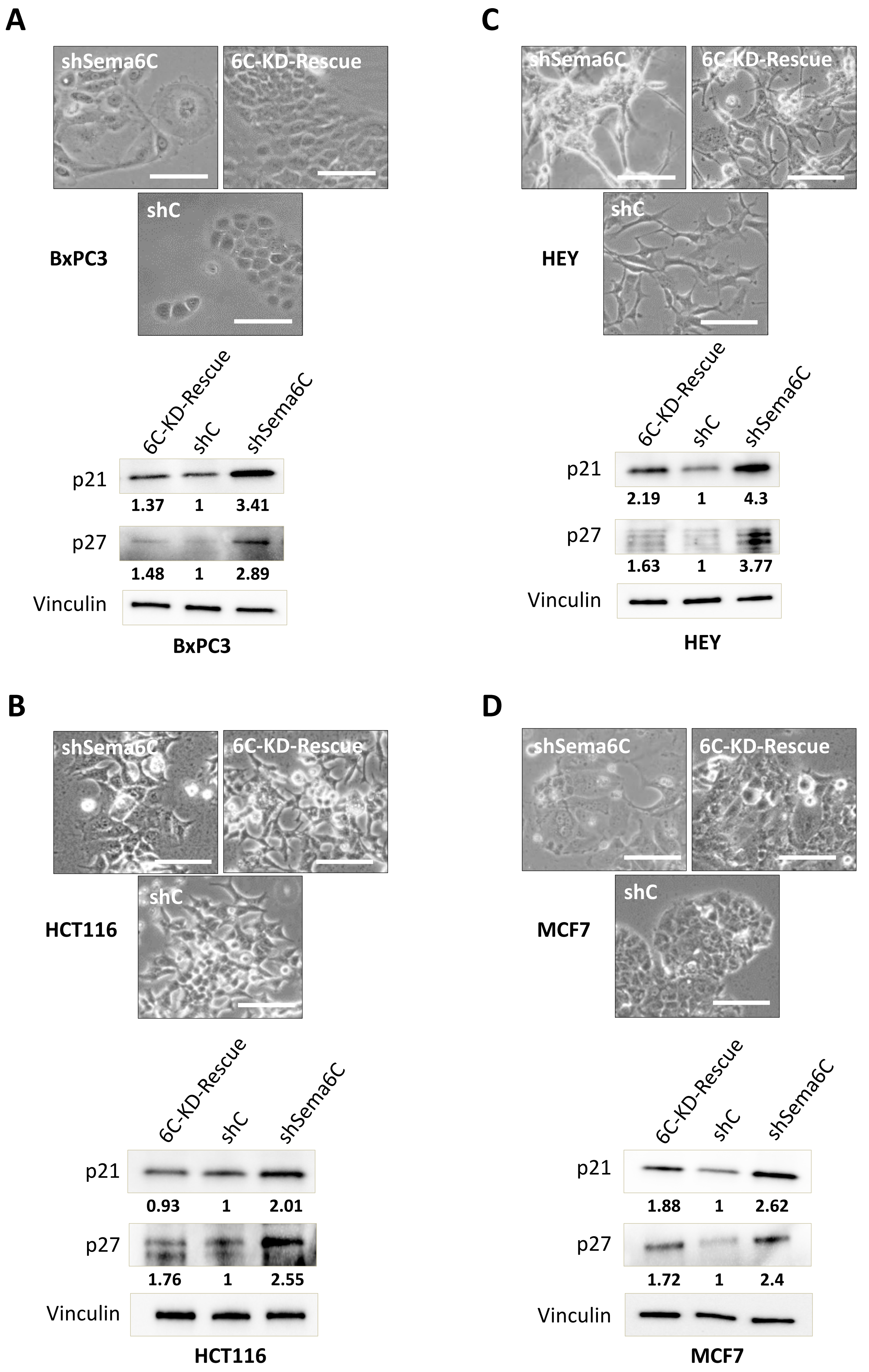
A
shSema6C
6C-KD-Rescue
shC
BxPC3
6C-KD-Rescue
shC
shSema6C
p21
1.37
1
3.41
p27
1.48
1
2.89
Vinculin
BxPC3
C
shSema6C
6C-KD-Rescue
shC
HEY
6C-KD-Rescue
shC
shSema6C
p21
2.19
1
4.3
p27
1.63
1
3.77
Vinculin
HEY
B
shSema6C
6C-KD-Rescue
shC
HCT116
6C-KD-Rescue
shC
shSema6C
p21
0.93
1
2.01
p27
1.76
1
2.55
Vinculin
HCT116
D
shSema6C
6C-KD-Rescue
shC
MCF7
6C-KD-Rescue
shC
shSema6C
p21
1.88
1
2.62
p27
1.72
1
2.4
Vinculin
MCF7

Suppl. Fig. 3

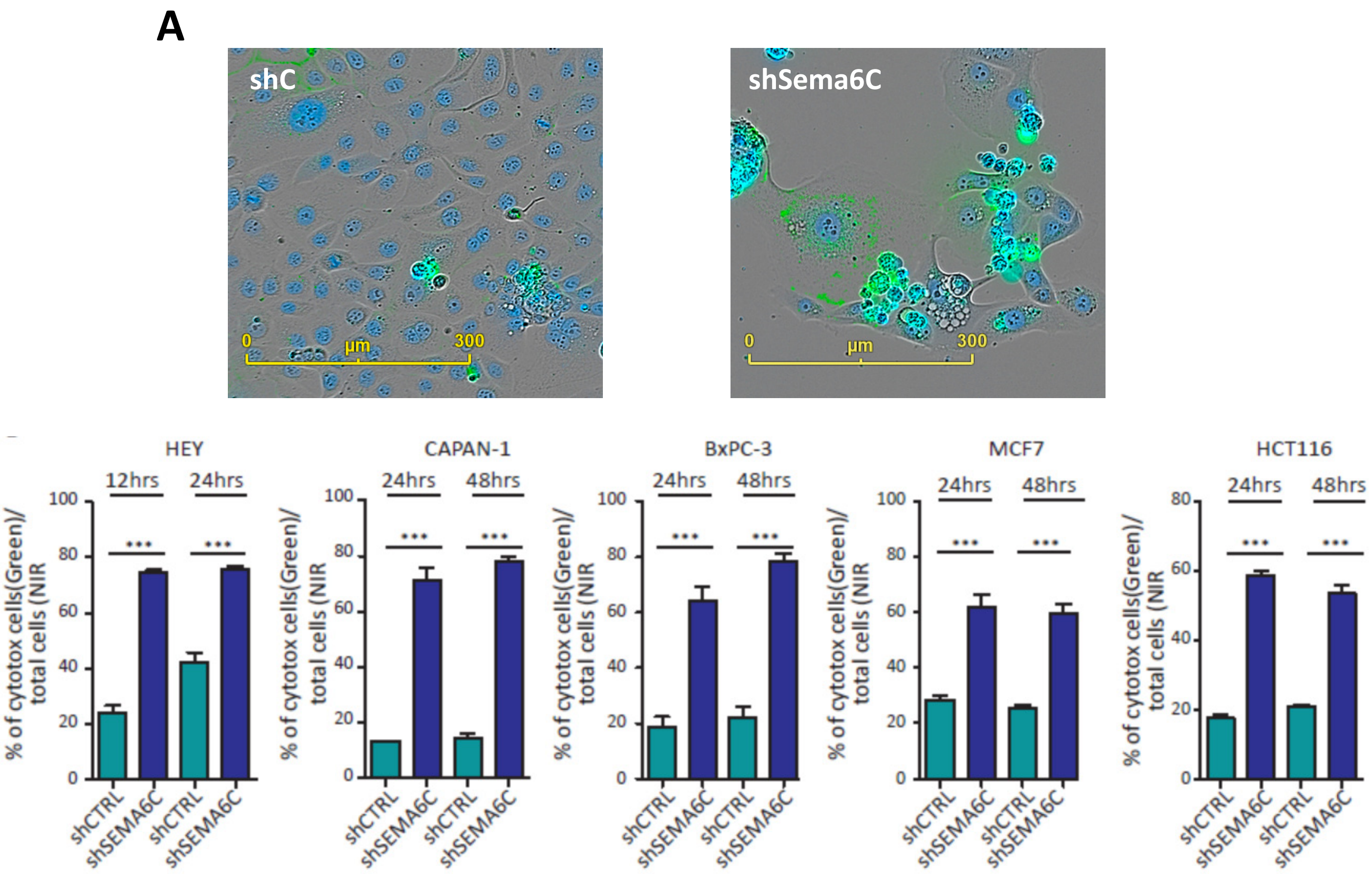

A
shC
0
µm
300
shSema6C
0
µm
300
HEY
12hrs
24hrs
CAPAN-1
24hrs
48hrs
BxPC-3
24hrs
48hrs
MCF7
24hrs
48hrs
HCT116
24hrs
48hrs
% of cytotox cells(Green)/ total cells (NIR
***
shCTRL
shSEMA6C

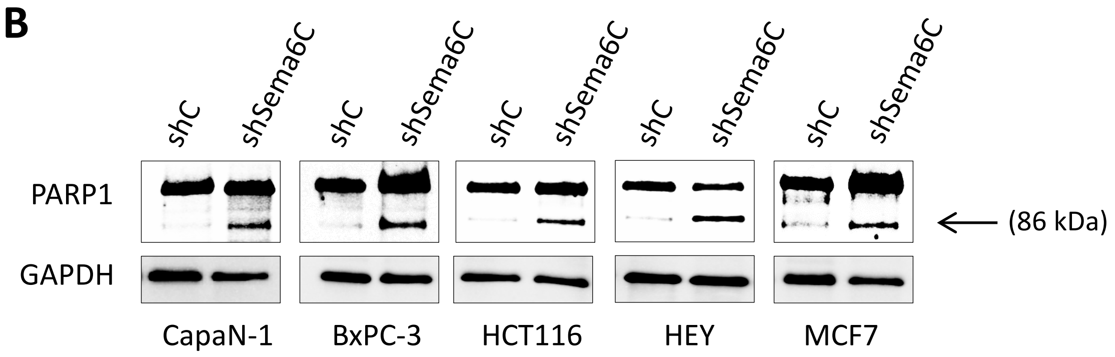

B
shC
shSema6C
PARP1
GAPDH
(86 kDa)
CapaN-1
BxPC-3
HCT116
HEY
MCF7

Supp. Fig. 4

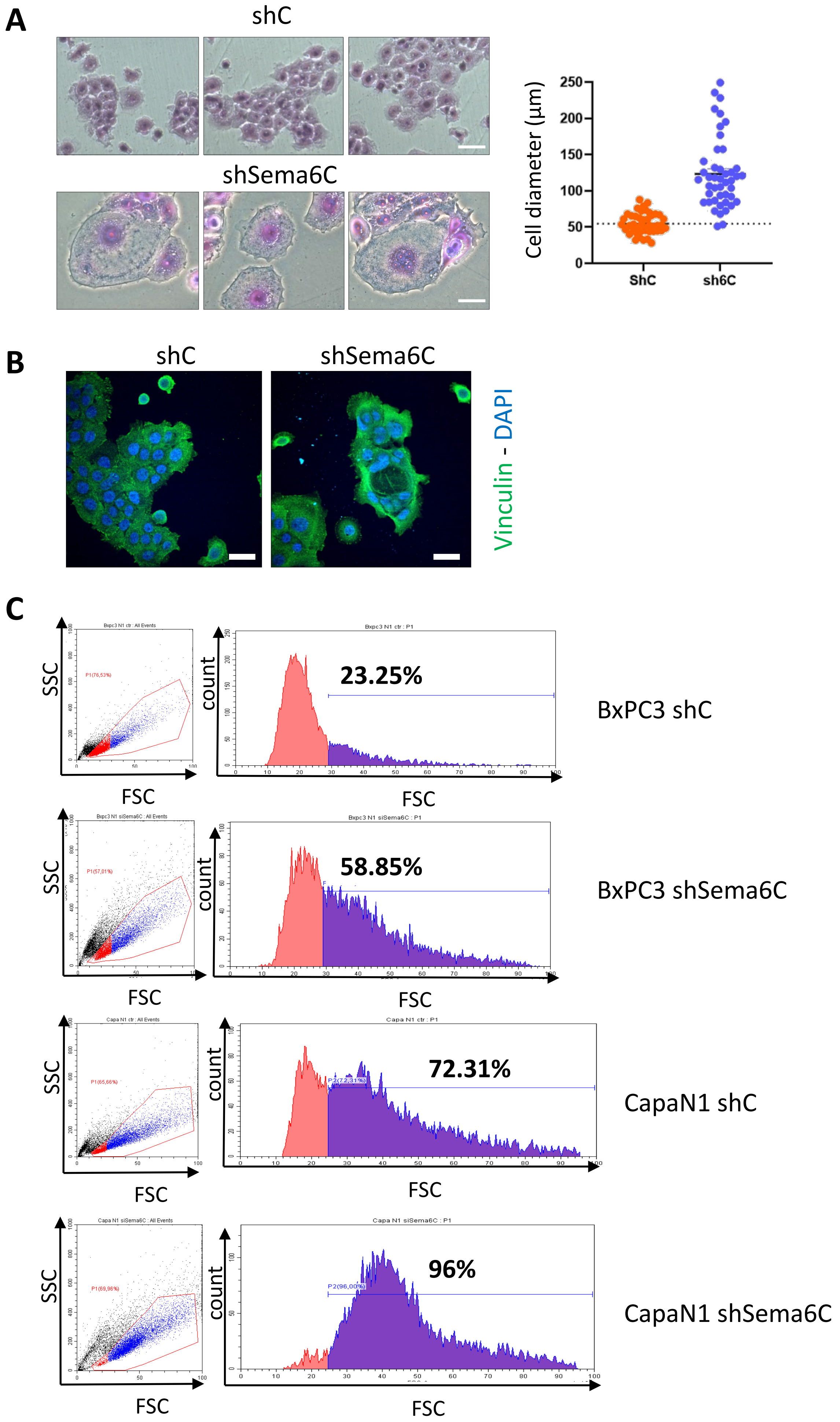
A
shC
shSema6C
Cell diameter (µm)
250
200
150
100
50
0
ShC
sh6C
B
shC
shSema6C
Vinculin - DAPI
C
SSC
FSC
count
23.25%
BxPC3 shC
58.85%
BxPC3 shSema6C
72.31%
CapaN1 shC
96%
CapaN1 shSema6C

Suppl. Fig. 5

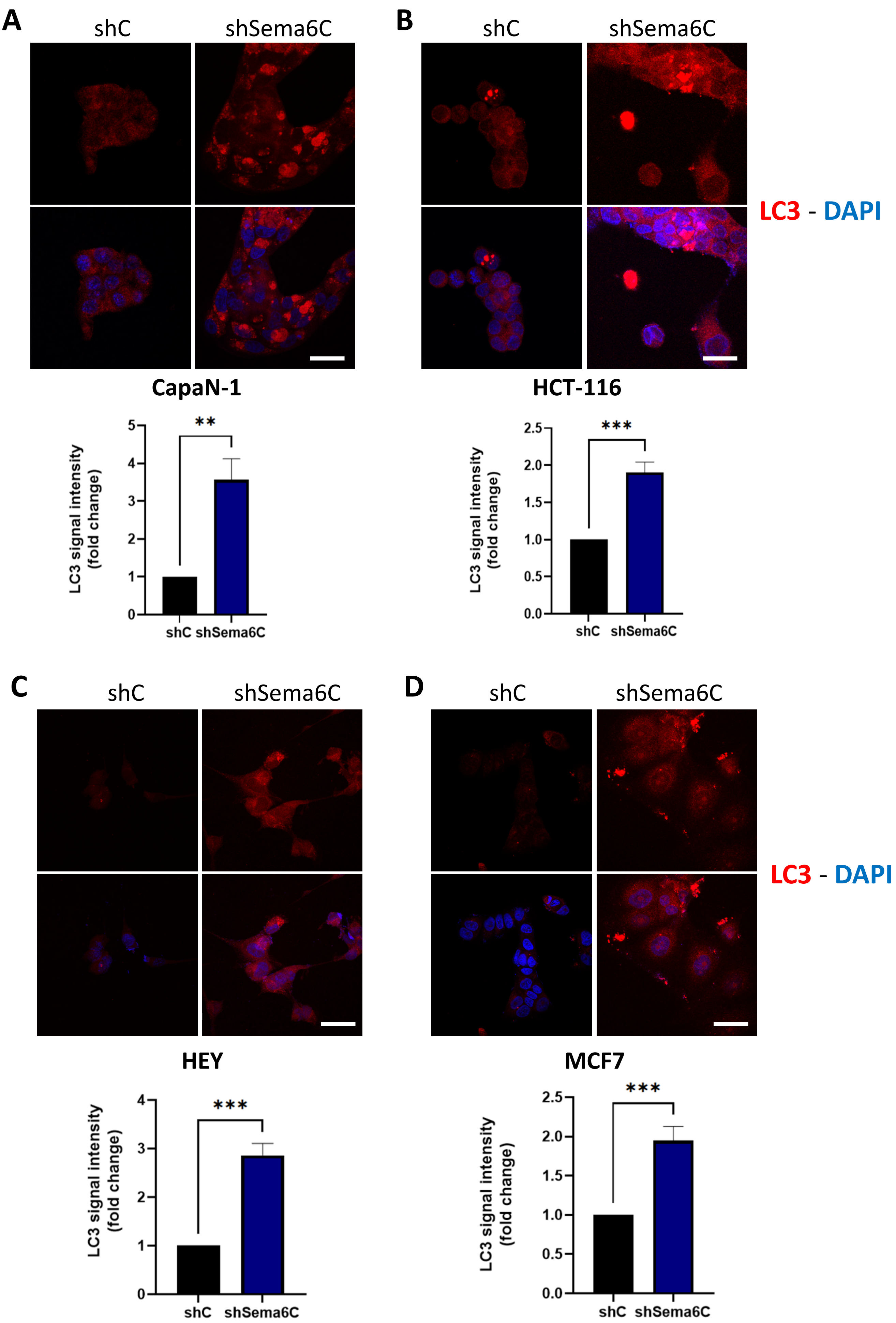
A
shC
shSema6C
CapaN-1
LC3 signal intensity (fold change)
**
shC shSema6C
B
shC
shSema6C
LC3 - DAPI
HCT-116
LC3 signal intensity (fold change)
***
shC shSema6C
C
shC
shSema6C
HEY
LC3 signal intensity (fold change)
***
shC shSema6C
D
shC
shSema6C
LC3 - DAPI
MCF7
LC3 signal intensity (fold change)
***
shC shSema6C

Suppl. Fig. 6

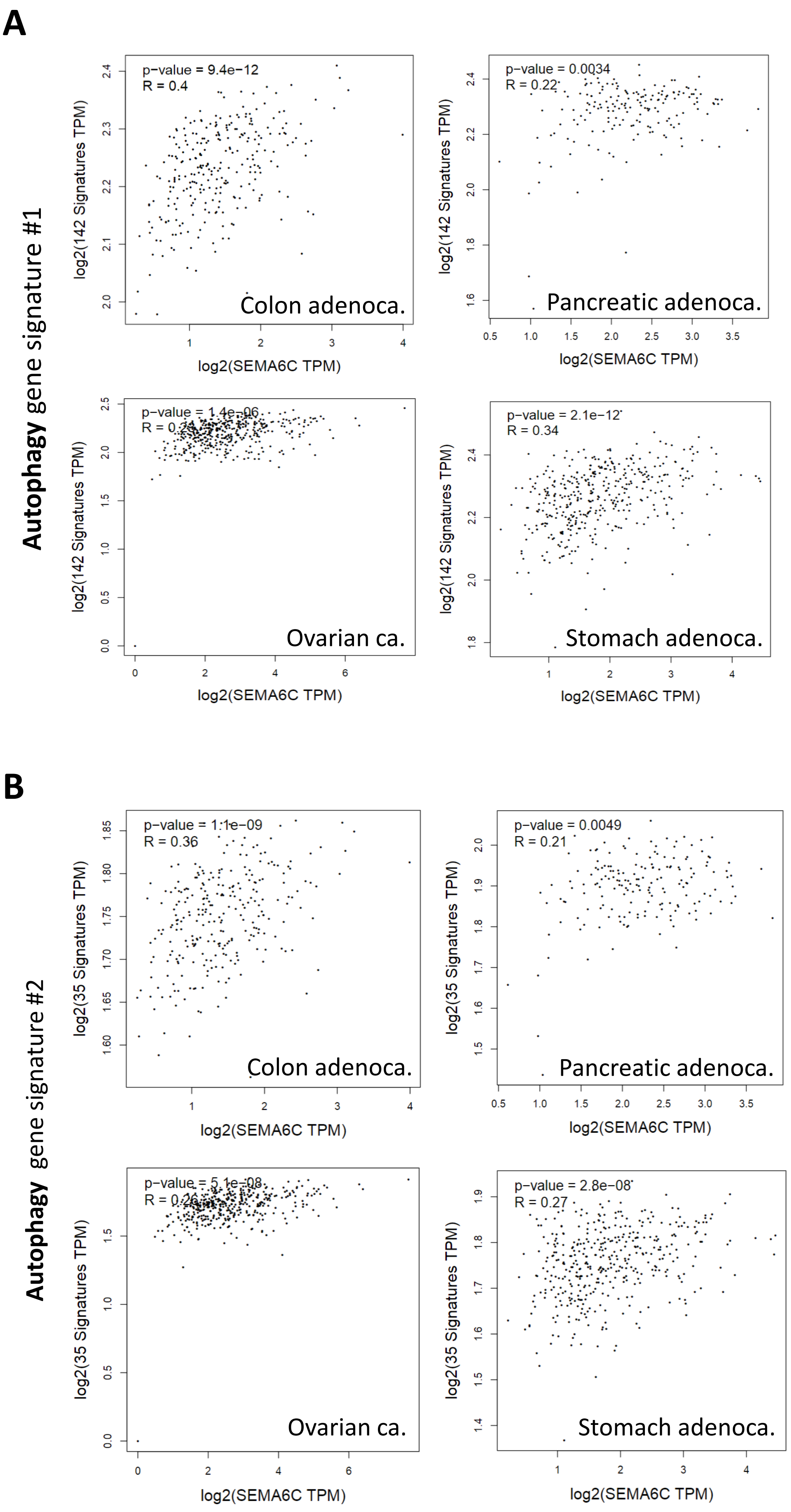
A
Autophagy gene signature #1
p−value = 9.4e−12
R = 0.4
Colon adenoca.
log2(142 Signatures TPM)
log2(SEMA6C TPM)
p−value = 0.0034
R = 0.22
Pancreatic adenoca.
p−value = 1.4e−06
R = 0.24
Ovarian ca.
p−value = 2.1e−12
R = 0.34
Stomach adenoca.
B
Autophagy gene signature #2
p−value = 1.1e−09
R = 0.36
Colon adenoca.
log2(35 Signatures TPM)
p−value = 0.0049
R = 0.21
Pancreatic adenoca.
p−value = 5.1e−08
R = 0.26
Ovarian ca.
p−value = 2.8e−08
R = 0.27
Stomach adenoca.

Supp. Fig. 7

A

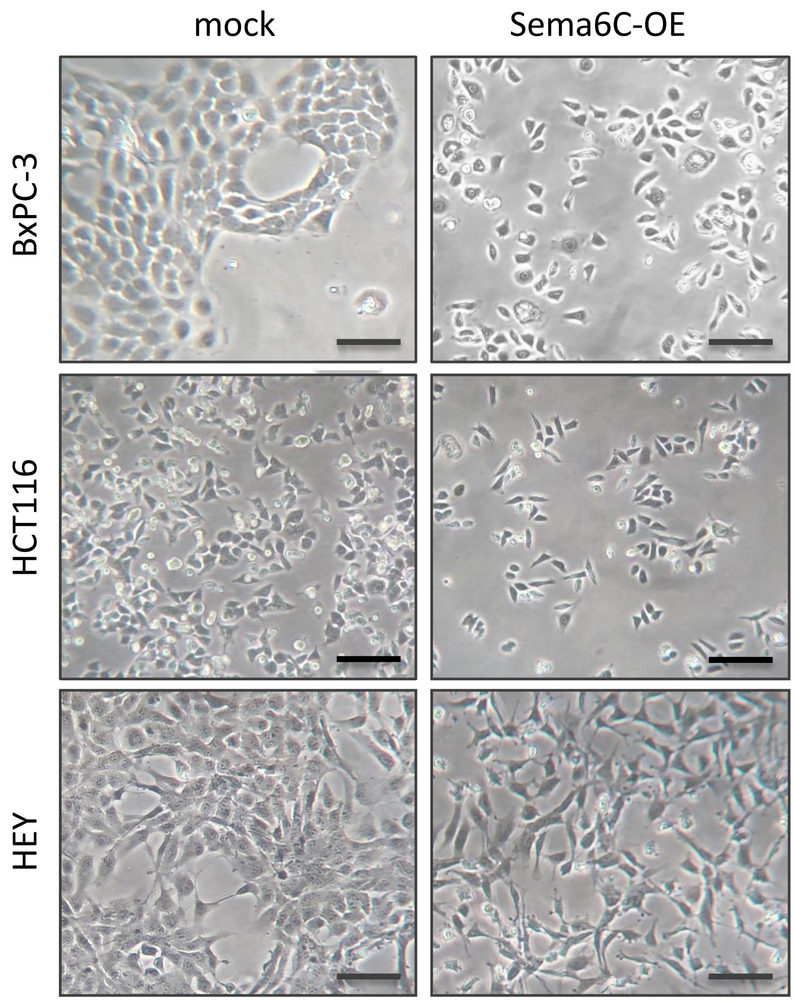

B

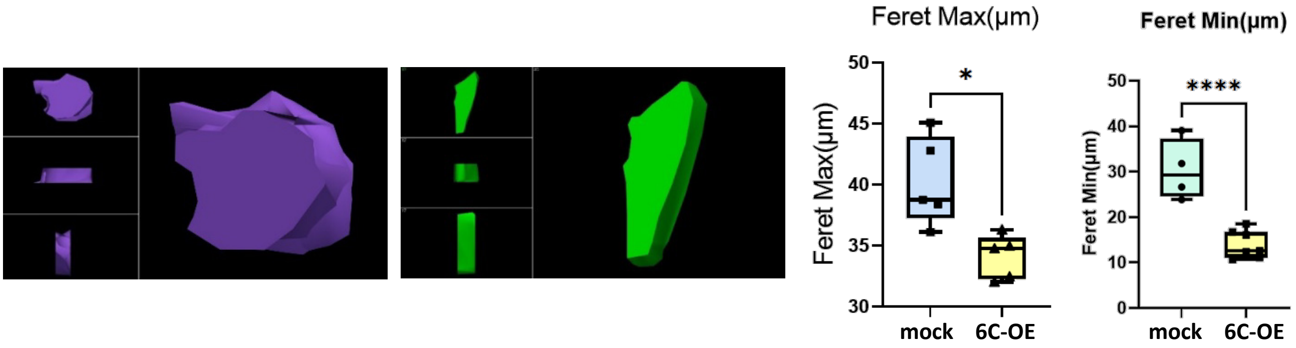

C

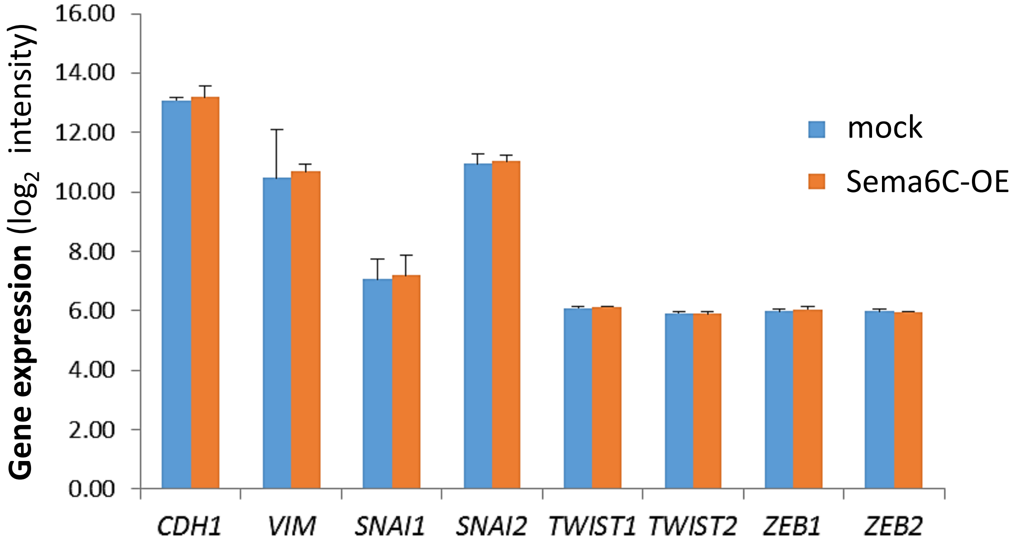

Supp. Fig. 8

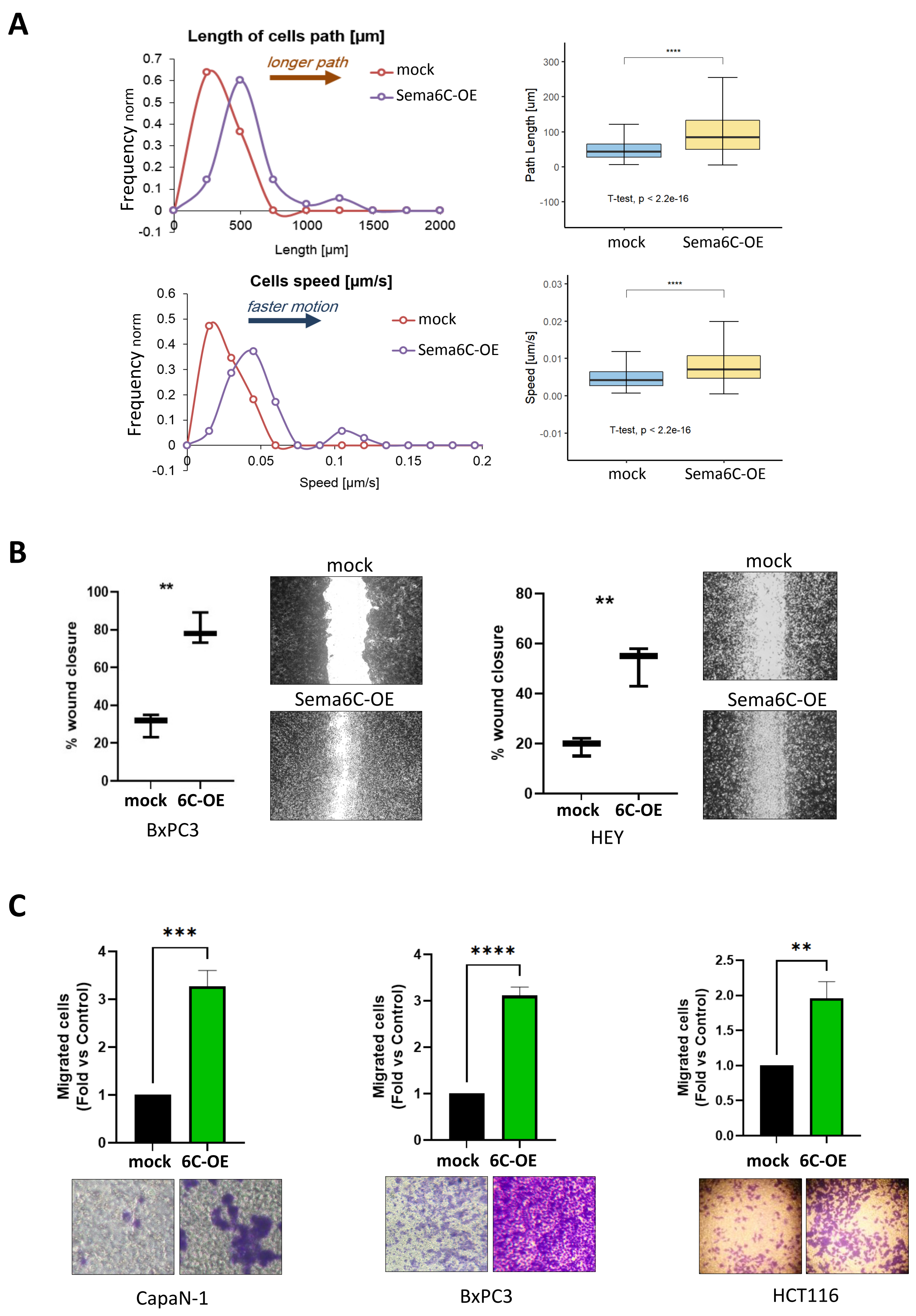

A
Length of cells path [μm]
longer path
mock
Sema6C-OE
Frequency norm
Length [μm]
****
Path Length [um]
T-test, p < 2.2e-16
mock
Sema6C-OE
Cells speed [μm/s]
faster motion
mock
Sema6C-OE
Frequency norm
Speed [μm/s]
****
Speed [um/s]
T-test, p < 2.2e-16
mock
Sema6C-OE
B
**
% wound closure
mock
6C-OE
BxPC3
mock
Sema6C-OE
**
% wound closure
mock
6C-OE
HEY
mock
Sema6C-OE
C
***
Migrated cells (Fold vs Control)
mock
6C-OE
CapaN-1
****
Migrated cells (Fold vs Control)
mock
6C-OE
BxPC3
**
Migrated cells (Fold vs Control)
mock
6C-OE
HCT116

Supp. Fig. 9

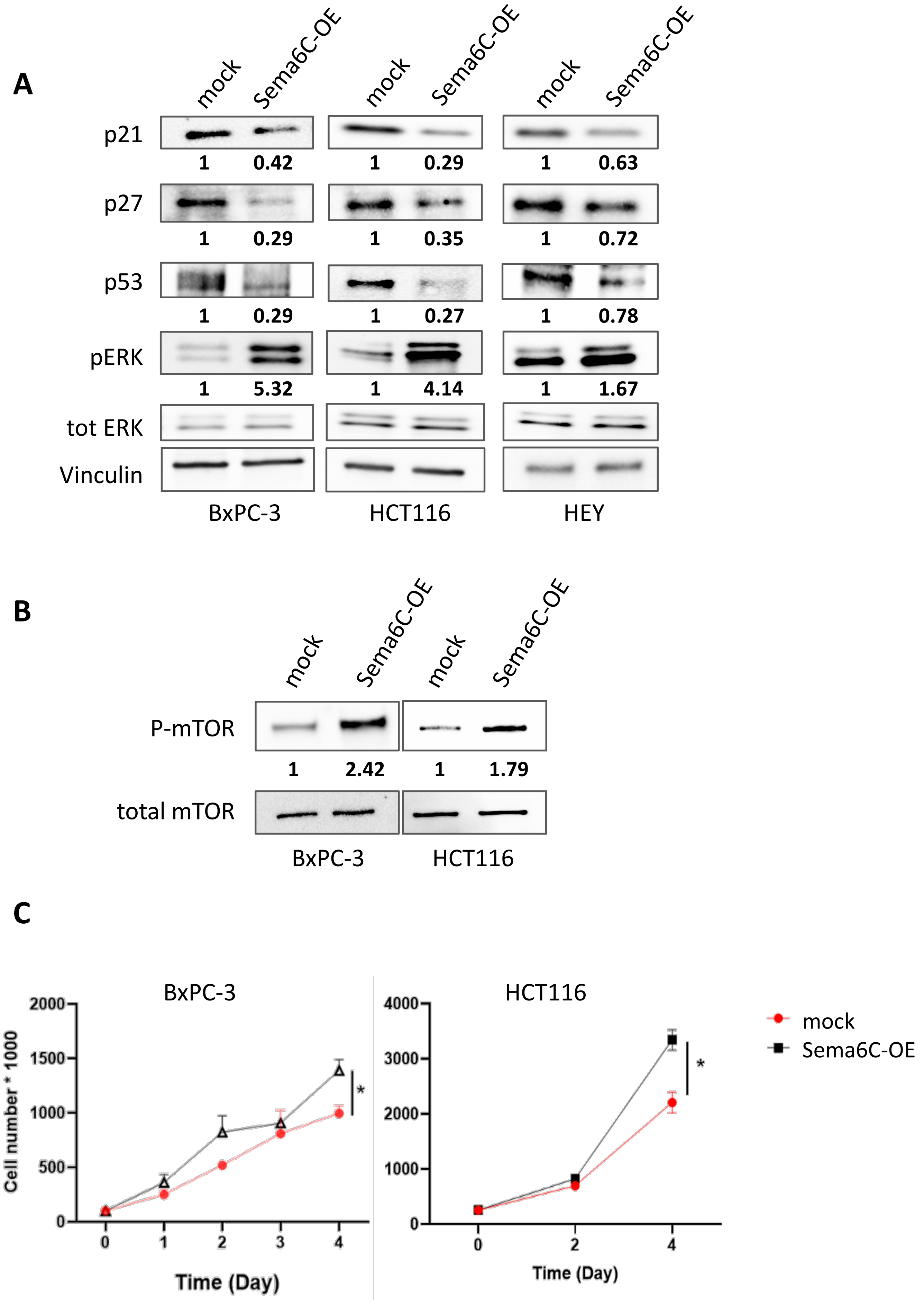
A
mock
Sema6C-OE
mock
Sema6C-OE
mock
Sema6C-OE
p21
1 0.42
1 0.29
1 0.63
p27
1 0.29
1 0.35
1 0.72
p53
1 0.29
1 0.27
1 0.78
pERK
1 5.32
1 4.14
1 1.67
tot ERK
Vinculin
BxPC-3
HCT116
HEY
B
mock
Sema6C-OE
mock
Sema6C-OE
P-mTOR
1 2.42
1 1.79
total mTOR
BxPC-3
HCT116
C
BxPC-3
Cell number * 1000
2000
1500
1000
500
0
0 1 2 3 4
Time (Day)
*
HCT116
4000
3000
2000
1000
0
0 2 4
Time (Day)
*
mock
Sema6C-OE

Supp. Fig. 10

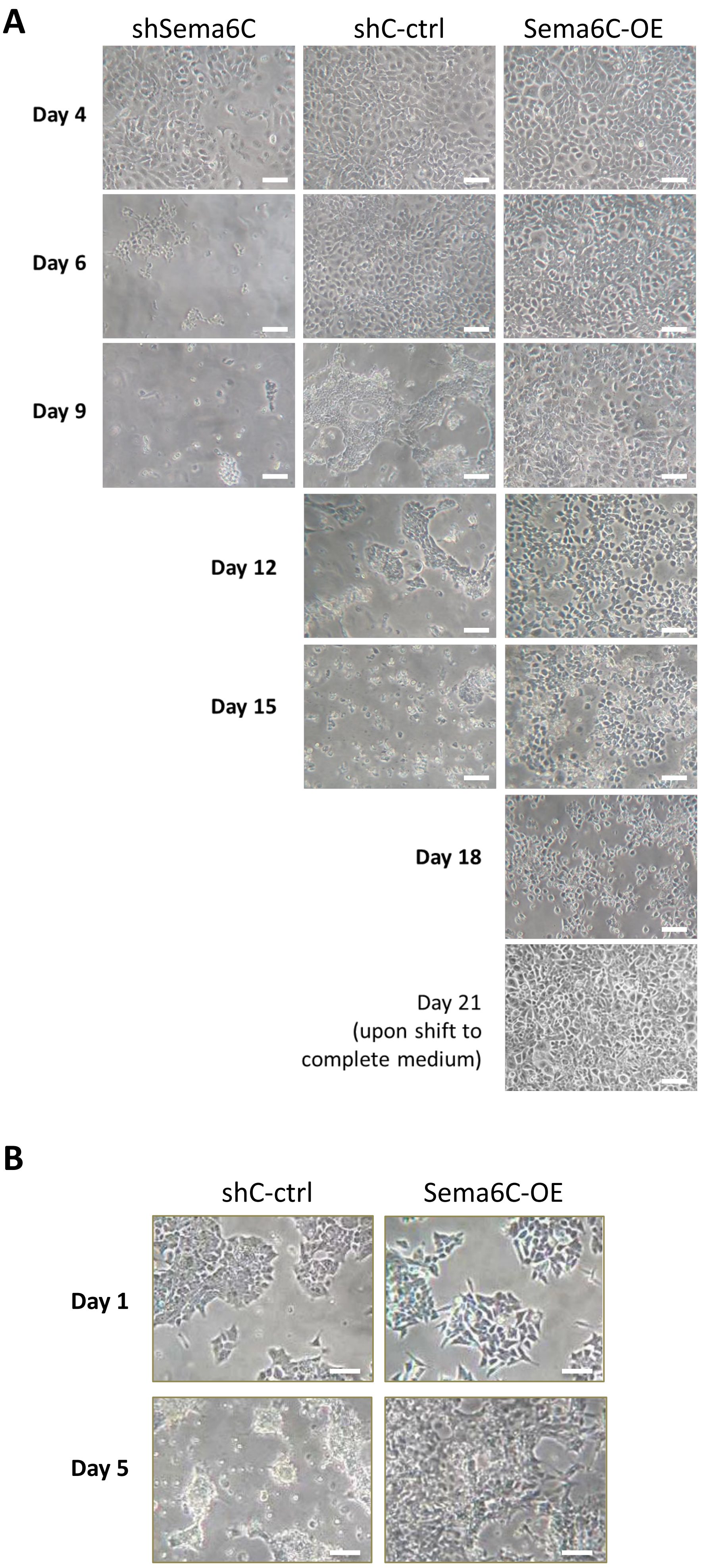
A
shSema6C
shC-ctrl
Sema6C-OE
Day 4
Day 6
Day 9
Day 12
Day 15
Day 18
Day 21
(upon shift to
complete medium)
B
shC-ctrl
Sema6C-OE
Day 1
Day 5

Supp. Fig. 11

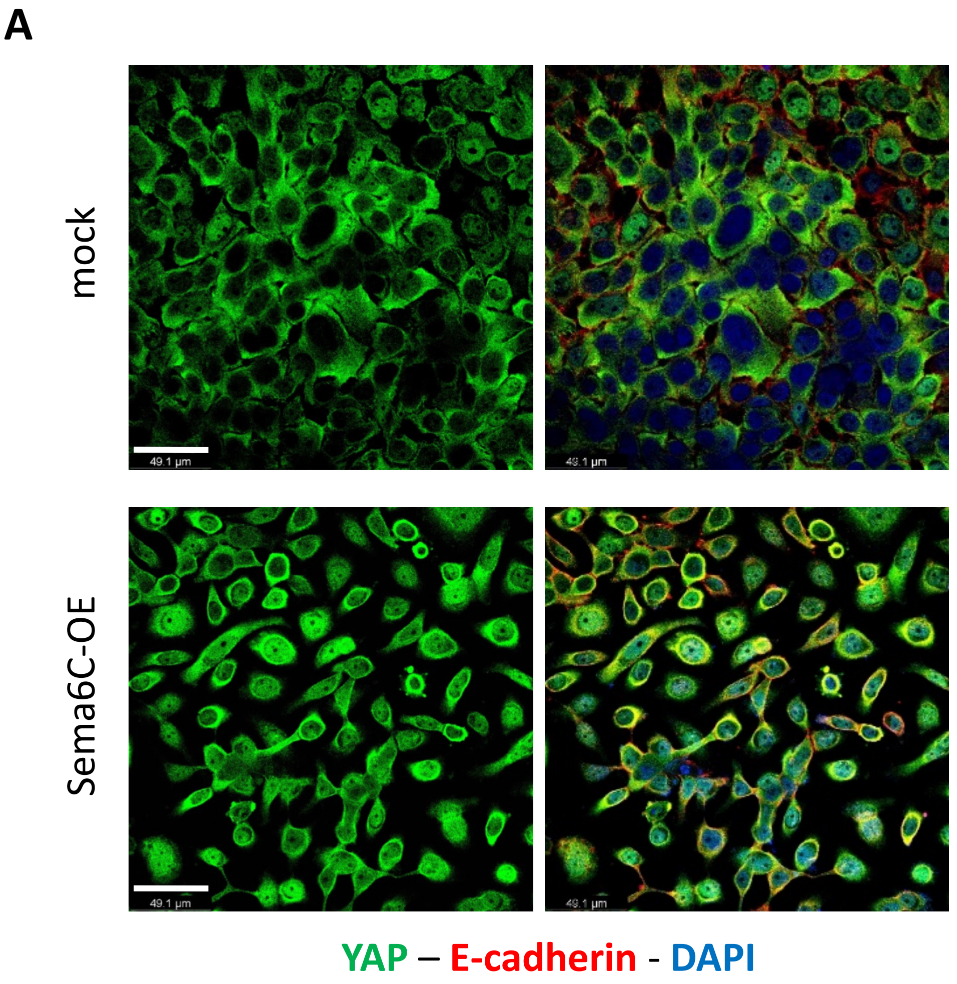
A
mock
Sema6C-OE
49.1 µm
49.1 µm
49.1 µm
49.1 µm
YAP – E-cadherin - DAPI

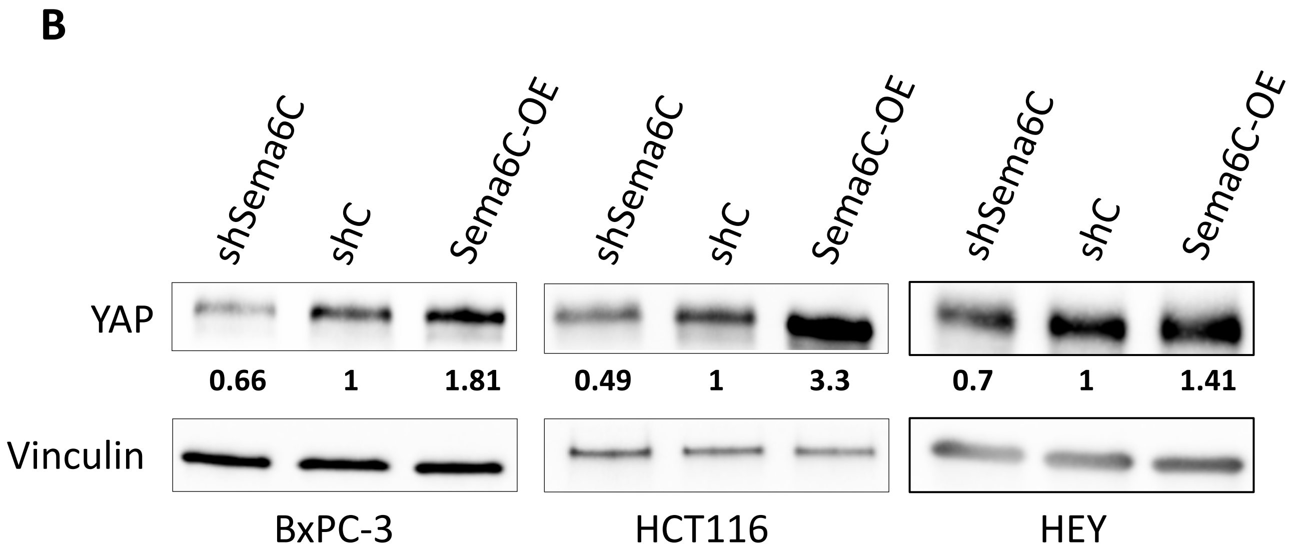
B
shSema6C
shC
Sema6C-OE
shSema6C
shC
Sema6C-OE
shSema6C
shC
Sema6C-OE
YAP
0.66
1
1.81
0.49
1
3.3
0.7
1
1.41
Vinculin
BxPC-3
HCT116
HEY

Supp. Fig. 12

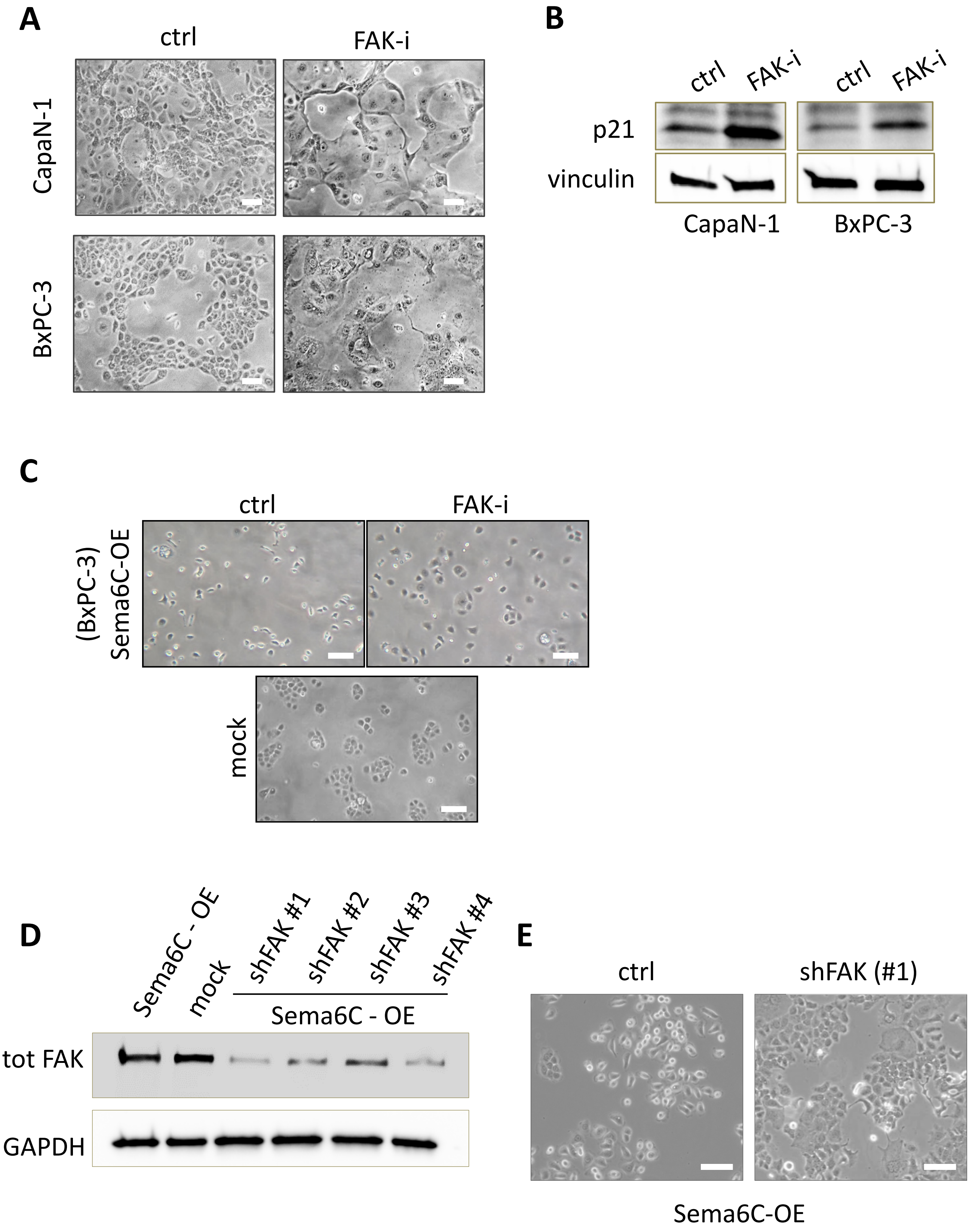
A
ctrl
FAK-i
CapaN-1
BxPC-3
B
ctrl
FAK-i
ctrl
FAK-i
p21
vinculin
CapaN-1
BxPC-3
C
ctrl
FAK-i
(BxPC-3)
Sema6C-OE
mock
D
Sema6C - OE
mock
shFAK #1
shFAK #2
shFAK #3
shFAK #4
Sema6C - OE
tot FAK
GAPDH
E
ctrl
shFAK (#1)
Sema6C-OE

Suppl. Fig. 13

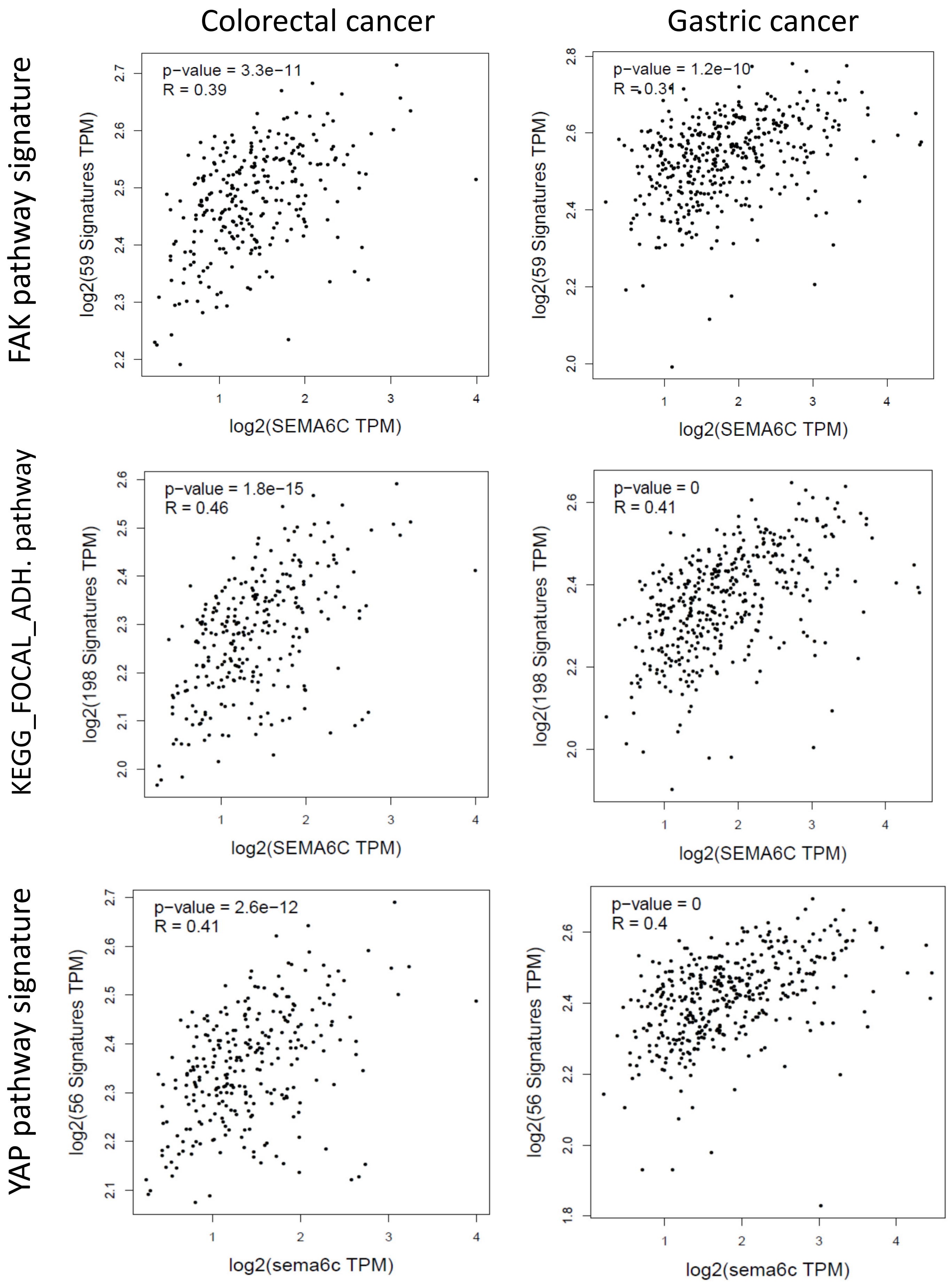
Colorectal cancer
Gastric cancer
FAK pathway signature
KEGG_FOCAL_ADH. pathway
YAP pathway signature
p−value = 3.3e−11
R = 0.39
log2(59 Signatures TPM)
log2(SEMA6C TPM)
p−value = 1.2e−10
R = 0.31
log2(59 Signatures TPM)
log2(SEMA6C TPM)
p−value = 1.8e−15
R = 0.46
log2(198 Signatures TPM)
log2(SEMA6C TPM)
p−value = 0
R = 0.41
log2(198 Signatures TPM)
log2(SEMA6C TPM)
p−value = 2.6e−12
R = 0.41
log2(56 Signatures TPM)
log2(sema6c TPM)
p−value = 0
R = 0.4
log2(56 Signatures TPM)
log2(sema6c TPM)

Suppl. Fig. 14

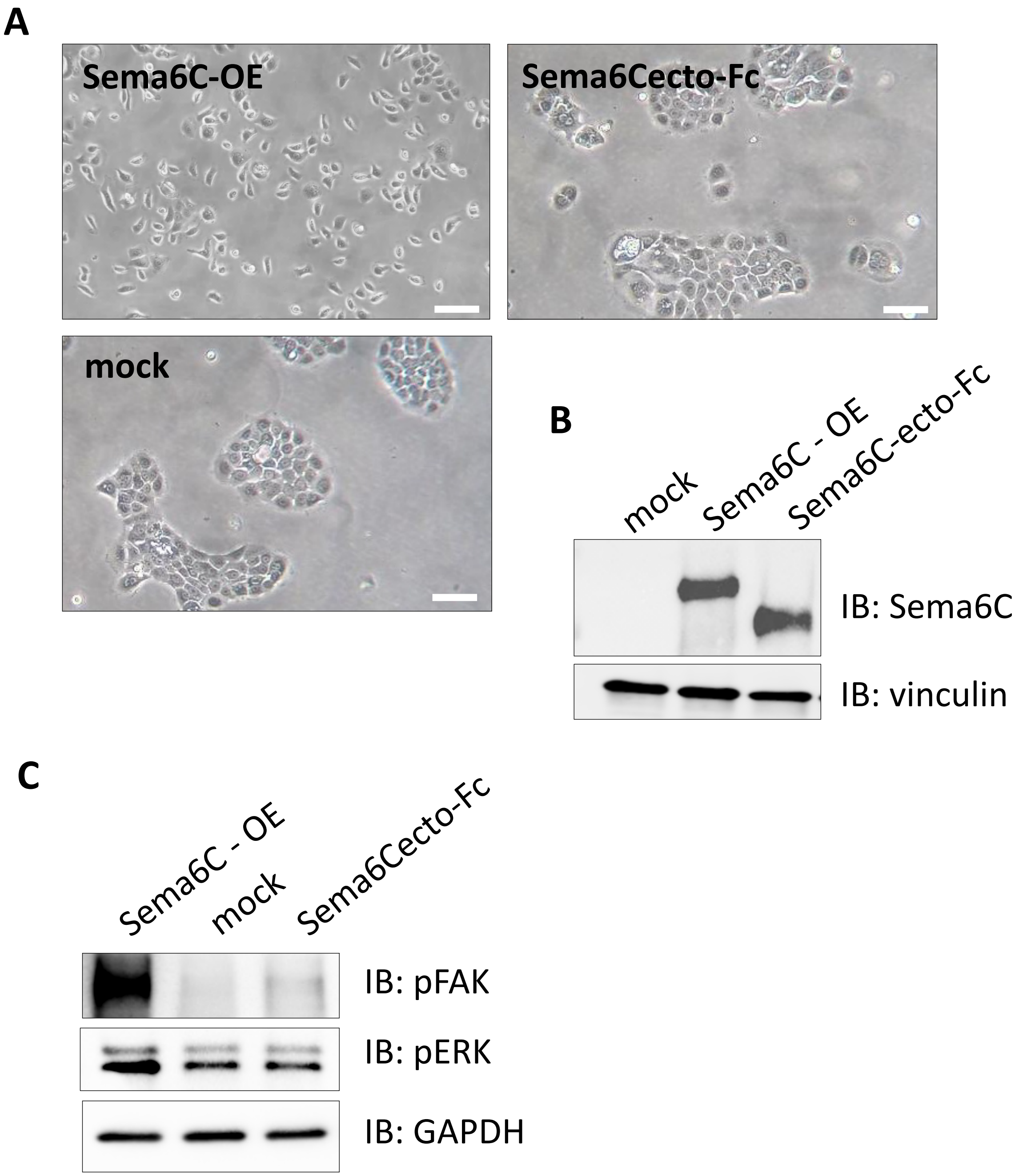
A
Sema6C-OE
Sema6Cecto-Fc
mock
B
mock
Sema6C - OE
Sema6C-ecto-Fc
IB: Sema6C
IB: vinculin
C
Sema6C - OE
mock
Sema6Cecto-Fc
IB: pFAK
IB: pERK
IB: GAPDH

Suppl. Fig. 15

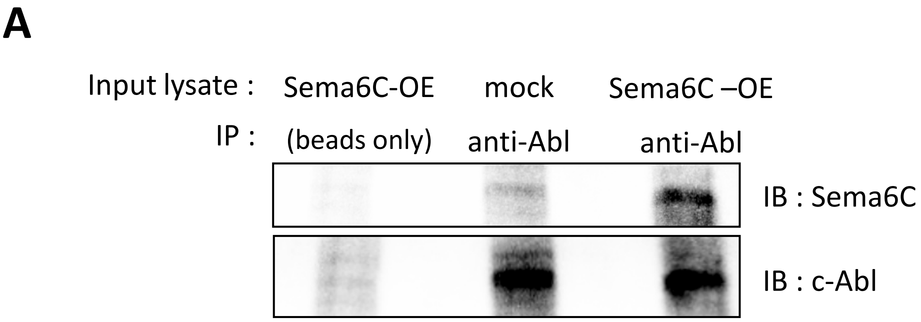
A
Input lysate :
Sema6C-OE
mock
Sema6C –OE
IP :
(beads only)
anti-Abl
anti-Abl
IB : Sema6C
IB : c-Abl

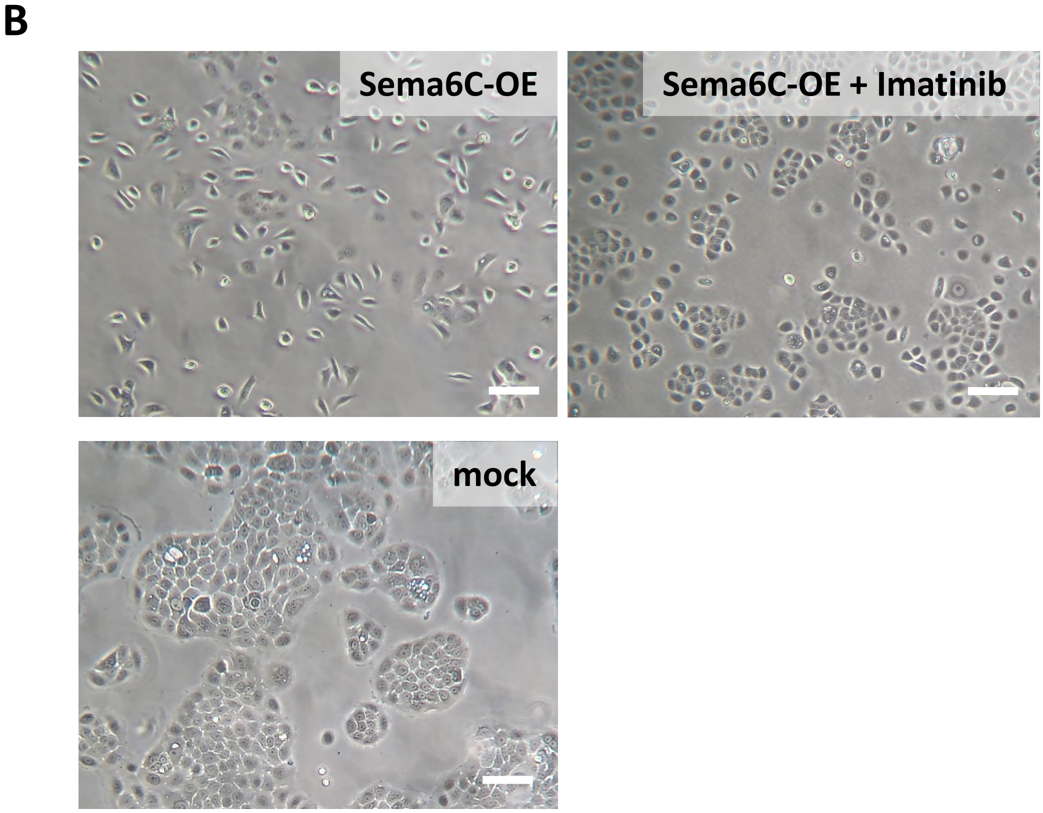
B
Sema6C-OE
Sema6C-OE + Imatinib
mock

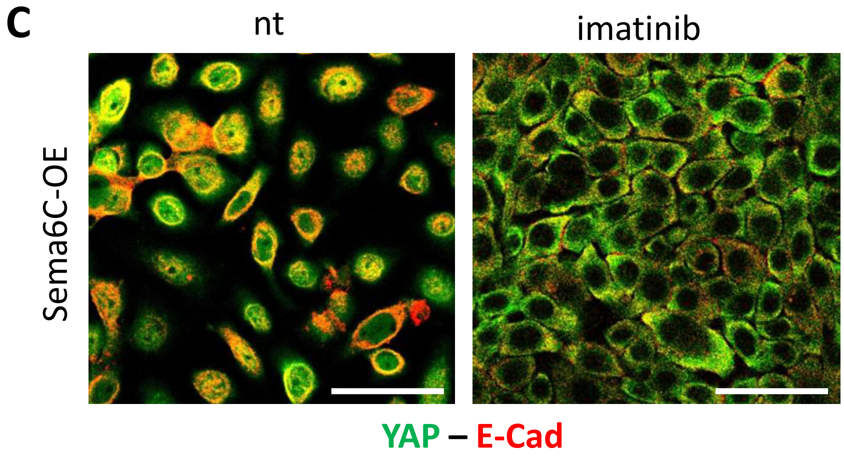
C
nt
imatinib
Sema6C-OE
YAP – E-Cad

LEGENDS TO SUPPLEMENTARY FIGURES

Suppl. Fig. 1. (A) Representative qPCR analysis of Sema6C expression in BxPC-3 cells subjected to Sema6C knock-down by targeted shRNAs. The graph shows average values ±SD (N=3); statistical analysis by unpaired t-test with Welch's correction: ***p < 0.001. **(B)** qPCR analysis of Sema6C expression in CapaN-1 cells subjected to Sema6C knock-down by two independent shRNA sequences (see Methods). shRNA sequence #1 was commonly used in all other experiments in this study, where not specified. The graph shows average values ±SD (N = 3 replicates/group); statistical analysis by one-way ANOVA: ***p < 0.001. **(C)** Representative phase contrast micrographs of the indicated cancer cell lines subjected to Sema6C knock-down by two independent shRNA sequences (as above), or controls transduced with empty vector (shC). **(D)** Western blotting analysis of Sema6C protein expression and p21/p27 cyclin-dependent kinase inhibitors in the same cells shown above. GAPDH levels provided a protein loading control and were used in the normalization of band intensity analyses; the indicated values represent fold-changes vs. respective shC controls.

Suppl. Fig. 2. (A) BxPC-3, **(B)** HCT116, **(C)** HEY, and **(D)** MCF7 cells subjected to Sema6C knock-down (shSema6C) were further transferred with a cDNA construct forcing Sema6C re-expression (6C-KD-Rescue); 72 hours later, representative phase contrast micrographs show reversion to a cellular phenotype comparable to control cells (shC, shown for reference). Scale bars: 100µm. Moreover, western blotting results (shown in each panel for the respective conditions) show reversion of p21/p27 upregulation upon Sema6C rescued expression. Vinculin levels provided a protein loading control, and were used in the normalization of band intensity analyses; the indicated values represent fold-changes vs. respective shC controls.

Suppl. Fig. 3. (A) In order to assess the potential cytotoxicity of Sema6C knock-down, engineered cancer cells and respective controls (as described in main Fig. 1) were labeled in culture with the Cytotox Green Dye, and with Nuclight Rapid NIR (to counterstain the nuclei of all adherent cells), as shown in two representative images on the top. IncuCyte device and analytical software were used to assess the fraction of dying cells, at two progressive time points, per each condition. The graphs show average values ±SD (N=3). Statistical analysis by one-way Anova with Bonferroni correction: p*< 0.05; p**< 0.01; p***< 0.001. **(B)** Western blotting analysis of PARP-1, and of its proteolytic product (arrow) indicative of apoptosis, in the indicated Sema6C-depleted and control cancer cells.

Suppl. Fig. 4. (A) Micrographs of crystal violet-stained BxPC3 cancer cells, either control or Sema6C-silenced, were subjected to morphometric analysis of cell diameters (by ImageJ); quantitative results are shown in the graph. Scale bars: 50µm. **(B)** Representative confocal images of Control and Sema6C-silenced cells immunostained to detect vinculin (green), while the nuclei were stained with DAPI (blue). Scale bars: 50µm. **(C)** Cytofluorimetric analysis of BxPC3 cancer cells, either control or Sema6C-silenced. In P1 were gated living cells based on the forward (FSC) and side scatter (SSC), while right panels shown FSC histograms of the cells gated in P1. Two overlapping populations with different cell size are distinguishable, and the threshold was defined based on FSC values in control cells; the percentage of large cells was then highlighted for each condition.

Suppl. Fig. 5. Representative confocal images of the autophagic protein marker LC3 (red) in control or Sema6C-silenced (A) (B) (C) (D) cancer cells; nuclei were stained by DAPI. Scale bars: 25µm The bar graphs at the bottom show the quantification of LC3 optical density fold-change upon Sema6C-depletion (average ± SD; N = 3 replicates/group). Unpaired t test with Welch's correction: $**p < 0.001$; $***p < 0.001$.

Suppl. Fig. 6. SEMA6C expression in human cancer samples (TCGA data) was correlated with GSEA molecular signatures: (#1, top) GOBP_Positive_Regulation_of_Autophagy (M15852), and (#2, bottom) KEGG_Regulation_of_Autophagy (M6382), by GEPIA-2 application (http://gepia2.cancer-pku.cn/#index).

Suppl. Fig. 7. (A) Phase contrast micrographs of diverse cancer cells, either control or transduced to overexpress Sema6C (Sema6C-OE). Scale bars: 50 µm. **(B)** Morphometric (Sholl) analysis of the cellular shape by Neurolucida software, applied to images of control or Sema6C-OE BxPC-3 (N = 5 replicates for group; 50 cells/replicate). Unpaired t test with Welch's correction: $**p < 0.01$; $****p<0.0001$. **(C)** The graph shows the mRNA expression analysis of the indicated genes associated with classical Epithelial-Mesenchymal Transition (EMT), in BxPC-3 cells, either control or Sema6C-OE. (N=3 replicates/group). No statistically significant variations were detected.

Suppl. Fig. 8. (A) The spontaneous motility of fluorescently labeled BxPC-3 cells, either Sema6C-overexpressing(OE) or mock controls, was analyzed in culture by time-lapse imaging with a confocal microscope (see Methods for details). Images were taken from each XY position of the array every 3 minutes and 9 seconds for a total time-lapse acquisition of 4 hours (see **Suppl. Movies 1 and 2**). Cells movements were analysed using the Cell Motility plugin of NIS-Elements software. The average speed and the average path length of 50 randomly selected cells from each sample were evaluated and compared. The normalized distribution of path length [µm] and speed [µm/s] is reported for mock control cells (red) and Sema6C-overexpressing(OE) cells (blue), respectively. The shift towards a longer path and a faster motion of Sema6C-OE cells, appreciable in the curve on the left, is further quantified in the box plot on the right. The graphs reveal a statistically significant increase (assessed by Student's t-test: $****p < 0.0001$) in both path length and speed of Sema6C-OE cells (yellow) with respect to controls (blue). **(B)** The migration of the indicated cancer cells, either Sema6C-OE or mock controls, was analyzed by wound healing assay (see Methods for details). The box blots show % of wound closure quantified after 16 hours from scratching the cell monolayer. Representative images are show on the left. The statistical significance was analyzed by T-test: $**p<0.01$. **(C)** Individual cell migration across 8 µm pores of the semipermeable membrane of Transwell inserts was quantified as described in Methods. The graphs show average motility fold-change (± SD) of the indicated Sema6C-OE cancer cells, vs. respective controls. The statistical significance was analyzed by T-test: $**p<0.01$; $***p<0.001$; $****p < 0.0001$.

Suppl. Fig. 9. (A) Western blotting analysis and densitometric values of the expression of cyclin-dependent kinase inhibitors p21, p27 and p53, and of phosphorylated ERK levels, in multiple control

and Sema6C-silenced cancer cells. Vinculin and total ERK levels provided a protein loading control, and were used in the normalization of band intensity analyses for CDK-Is and pERK, respectively. The indicated values represent fold-changes in Sema6C-silenced cells vs. each respective control. **(B)** Western blotting analysis and densitometric values of phosphorylated mTOR levels in control and Sema6C-silenced cancer cells. Total mTOR levels provided a protein loading control and were used in the normalization of band intensity analyses; the indicated values represent fold-changes in Sema6C-silenced cells vs. each respective control. **(C)** Growth curve analysis of BxPC-3 (a) and HCT-116 (b) cancer cells, either control or Sema6C-overexpressing; (N=3 replicates/group). Two-way RM ANOVA. $^*p < 0.05$.

Suppl. Fig. 10. (A) Representative images of Sema6C-silenced, control, and Sema6C-overexpressing BxPC-3 cells, maintained in culture at confluence in serum-free conditions. The number of surviving cells in each condition was periodically scored and normalized counts are shown in main Fig. 3C. At day 18, Sema6C-OE cells were the only survivors; upon switching to fresh FBS-containing medium, these cells resumed growth reaching confluence by day 21. Scale bars: 50µm. **(B)** Representative images of HCT116 cells, either control or Sema6C-OE, subjected to a similar experiment as above. These cells were more susceptible to starvation, and controls started to succumb at an earlier stage compared to pancreatic cancer cells above. Scale bars: 50µm.

Suppl. Fig. 11. (A) Representative confocal images of YAP (green), E-cadherin (red) immunofluorescence showing the subcellular localization in control and Sema6C-overexpressing BxPC-3 cells maintained in serum-free medium. Nuclei were counterstained by DAPI; scale bars: 50µm. **(B)** Western blotting analysis and densitometric values of YAP protein levels in multiple cancer cells, either Sema6C-silenced, Sema6C-overexpressing, or controls. Vinculin levels provided a protein loading control, and were used in the normalization of band intensity analyses; the indicated values represent fold-changes in Sema6C-modulated cells vs. each respective control.

Suppl. Fig. 12. (A) Phase contrast micrographs of cancer cells subjected (or not) to treatment with the selective FAK-inhibitor PF-573228 (5µM) for 48 hours. Scale bar: 50 µm. The induced phenotype closely resembles that caused by Sema6C knock-down. **(B)** Western blotting analysis of p21 CDK-I levels in the same cells shown in the previous panel; vinculin provided a protein loading control. **(C)** Phase contrast micrographs of Sema6C-overexpressing BxPC-3 upon treatment with the selective FAK-inhibitor PF-573228 (5 µM) or vehicle alone (ctrl), for 1 hour. Untreated mock-transduced control cells are shown below, for comparison. Scale bars: 50µm. **(D)** Western blotting analysis of FAK expression in BxPC-3 cell subjected to gene knock-down by expression of four independent targeted shRNAs (see Methods; sequence #1 was used in other experiments shown in this paper). **(E)** Phase contrast micrographs of Sema6C-overexpressing BxPC-3 cells subjected, or not, to FAK knock-down by expression of shRNA sequence #1 (described above). Scale bars: 50µm.

Suppl. Fig. 13. SEMA6C expression in human cancer samples (TCGA data) was correlated with GSEA molecular signatures: PID_FAK_Pathway (M281), KEGG_Focal_Adhesion Pathway (M7253), and Cordenonsi_YAP_conserved_signature pathway (M2871), by GEPIA-2 application (http://gepia2.cancer-pku.cn/#index).

Suppl. Fig. 14. (A) Phase contrast micrographs of BxPC-3 cells stably overexpressing either full-length Sema6C, or Sema6C extracellular domain fused to Ig-Fc (Sema6Cecto-Fc), or mock-transfected; scale bar=50µm. **(B)** Western blotting analysis of Sema6C expression in the cells shown above; vinculin provided a protein loading control. **(C)** Western blotting analysis of pFAK and pERK levels in the same cells shown in the previous panel; GAPDH provided a protein loading control. The image is representative of three independent experiments showing consistent results.

Suppl. Fig. 15. (A) Co-immunoprecipitation experiments performed by incubating protein lysates of Sema6C-OE or endogenously expressing BxPC-3 control cells with anti-Abl coated beads (or beads only); the separated immunocomplexes were then probed to detect the presence of c-Abl kinase and any associated Sema6C protein. Data are representative of two independent experiments with consistent results. **(B)** Phase contrast micrographs of Sema6C-OE BxPC-3 cells in basal conditions and upon treatment with the Abl inhibitor Imatinib (10 µM) for 2 hours. Untreated mock-transduced control cells are shown below, for comparison. Scale bars: 50µm. **(C)** Representative confocal images of YAP (green) and E-cadherin (red) immunofluorescence showing the re-localization of YAP in the cytoplasm in Sema6C-overexpressing BxPC-3 cells treated with 10 µM imatinib for 2 hours. Scale bars: 50 µm.
